# Multimodal Profiling of Complement Expression and Association With Outcomes in ANCA-Associated Vasculitis With Glomerulonephritis

**DOI:** 10.1016/j.ekir.2026.107056

**Published:** 2026-08-26

**Authors:** Benoît Brilland, Victor Milon, Cyrille Vandenbussche, Nathalie Merillon, Eleonore Ourouda, Andrea Boizard-Moracchini, Maëva Roy, Odile Blanchet, Emeline Vinatier, Céline Beauvillain, Thomas Quéméneur, Assia Djema, Nicolas Henry, Giorgina Barbara Piccoli, Samuel Wacrenier, Yves Delneste, Patrick Blanco, David Langlais, Viviane Gnemmi, Marie-Christine Copin, Jean-François Augusto, RENVAS Registry Research Group, Franck Bourdon, Gérard Cardon, Maïté Daroux, Viviane Gnemmi, Thomas Guincestre, Maxime Hoffman, Zacharie Jomaa, Timothée Laboux, Céline Lebas, Valentine Quersin, Cyrille Vandenbussche, Nicolas Wayolle, Maine-Anjou Registry Research Group, Jean-François Augusto, Céline Beauvillain, Benoit Brilland, Jean-Philippe Coindre, Marie-Christine Copin, Maud Cousin, Anne Croué, Assia Djema, Fanny Guibert, Nicolas Henry, Giorgina Barbara Piccoli, Lise-Marie Pouteau, Clément Samoreau, Emeline Vinatier, Samuel Wacrenier

**Affiliations:** 1 Service de Néphrologie-Dialyse-Transplantation, CHU Angers, Angers, France; 2 Inserm, CNRS, CRCI2NA, Structure Fédérative de Recherche - Interactions Cellulaires et Applications Thérapeutiques (SFR ICAT), University of Angers, Nantes Université, Angers, France; 3 Department of Human Genetics, Victor Phillip Dahdaleh Institute of Genomic Medicine, McGill University, Montreal, Quebec, Canada; 4 Nephrology and Internal Medicine Department, Hospital of Valenciennes, Valenciennes, France; 5 Laboratoire d'Immunologie et d’Allergologie, CHU d’Angers, Angers, France; 6 Laboratoire d’Immunologie et Immunogénétique, FHU ACRONIM, Hôpital Pellegrin, Centre Hospitalier Universitaire de Bordeaux, Bordeaux, France; 7 Centre de Ressources Biologiques, CHU Angers, Angers, France; 8 Service de Néphrologie-Dialyse, Centre Hospitalier de Cholet, Cholet, France; 9 Service de Néphrologie-Dialyse, Centre Hospitalier de Laval, Laval, France; 10 Service de Néphrologie-Dialyse, Centre Hospitalier du Mans, Le Mans, France; 11 CNRS-UMR 5164, ImmunoConcept, Université de Bordeaux, Bordeaux, France; 12 Service d'anatomopathologie, Centre Hospitalier Universitaire de Lille, Lille, France; 13 U1352 BioPrev, University of Lille, Inserm, CHU Lille, JUNIA, Lille, France; 14 Département de pathologie cellulaire et tissulaire, CHU Angers, Angers, France

**Keywords:** ANCA, complement pathway, glomerulonephritis, outcomes, transcriptomic analyses, vasculitis

## Abstract

**Introduction:**

Antineutrophil cytoplasmic antibody (ANCA)-associated vasculitis (AAV) with glomerulonephritis (AAV-GN) is classically considered pauci-immune, yet complement activation is a central pathogenic mechanism. Whether intrarenal complement-related transcriptional programs are reflected by conventional serum or immunofluorescence markers, and whether they are clinically informative, remains unclear.

**Methods:**

In 369 patients with AAV-GN from 2 independent retrospective cohorts, complement was assessed across circulating, histopathologic, and transcriptomic layers (serum C3/C4, glomerular C1q/C3 deposition, and targeted kidney transcriptomics). Predefined classical and alternative pathway-related expression scores were derived from intrarenal gene expression. Associations with severity and outcomes were evaluated using multivariable Cox models. Public spatial transcriptomic data sets provided validation and localization.

**Results:**

Below-normal serum C3/C4 levels were uncommon. Low serum C3, but not C4, was associated with more severe kidney involvement, kidney failure (adjusted hazard ratio [aHR]: 2.0 [1.1–3.7]), and mortality (aHR: 2.3 [1.3–4.0]). Both glomerular C3 (47%) and C1q (11%) deposition identified more severe pathology and adverse outcomes. Compared with controls, AAV-GN kidneys showed increased complement-related expression—including classical pathway components, proximal alternative pathway genes (*C3*, *C5AR1)*, and regulatory transcripts—that was only weakly concordant with serum or deposition markers. Low alternative pathway-related expression was independently associated with better kidney (aHR: 0.3 [0.1–0.7]) and overall (aHR: 0.4 [0.2–0.9]) survival. Spatial transcriptomics validated these signatures and suggested contributions from both immune and resident kidney compartments.

**Conclusion:**

AAV-GN displays a reproducible intrarenal complement-related transcriptional program, only weakly captured by serum levels or routine immunofluorescence, associated with renal severity and outcomes. These findings support prospective studies integrating tissue complement transcriptomics for stratification and therapeutic hypothesis generation.

## Graphical abstract

**Figure:**
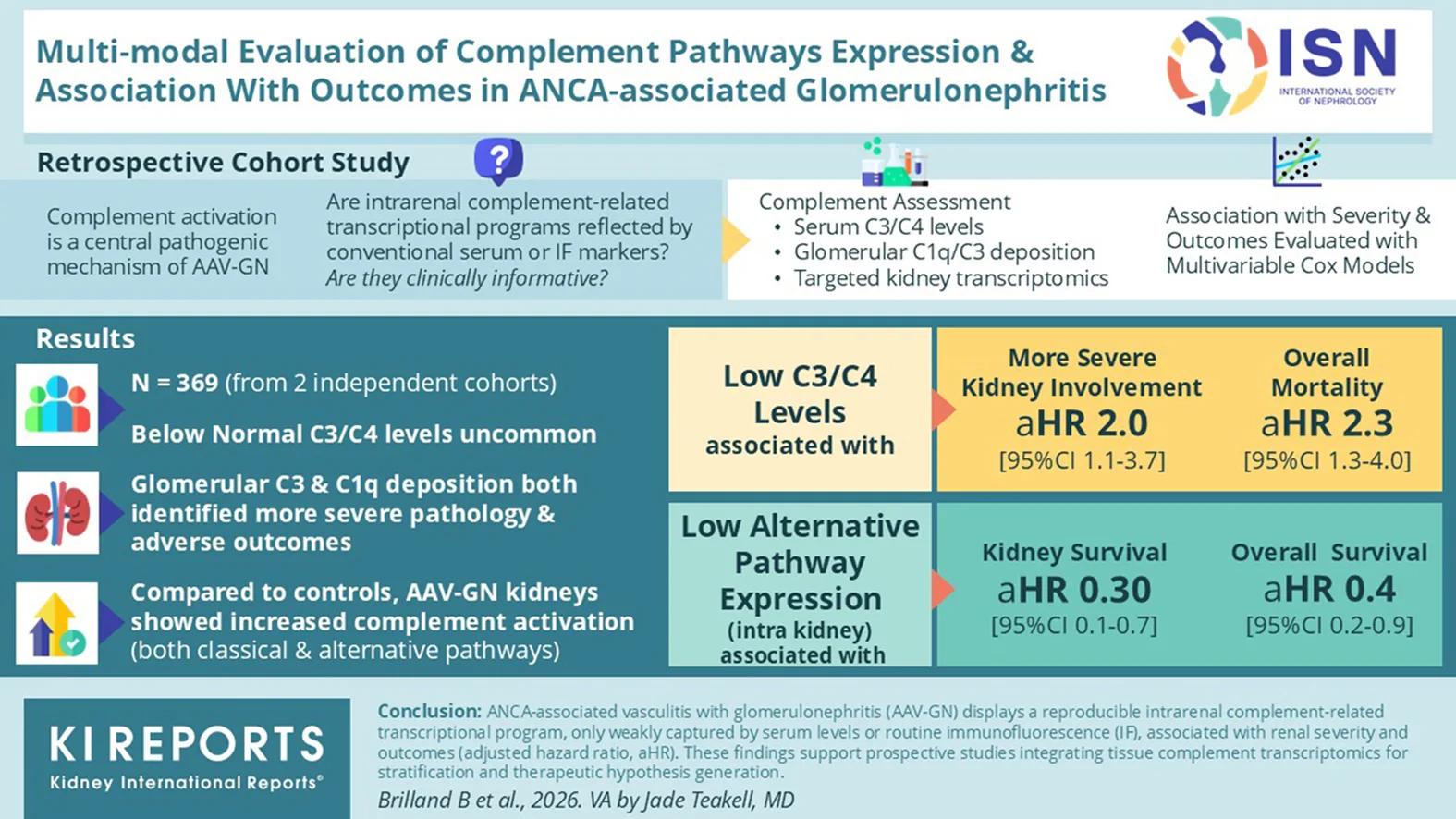

AAV are necrotizing small-vessel vasculitides in which renal involvement, manifesting as crescentic glomerulonephritis (AAV-GN), is a major determinant of long-term prognosis.^1^ Despite advances in immunosuppressive strategies, a substantial proportion of patients progress to kidney failure or relapse, highlighting the need for improved risk stratification and a deeper understanding of intrarenal disease mechanisms.^2^ Over the past 2 decades, the complement system has emerged as a key amplifier of ANCA-mediated inflammation,^3^ with a central role for the alternative pathway and the C5a-C5aR axis.^4^

However, the contribution of complement in human AAV-GN remains incompletely characterized. In clinical practice and observational studies, complement involvement is most often assessed using circulating C3/C4 levels and glomerular C1q/C3 deposition by immunofluorescence. Although AAV-GN is classically described as pauci-immune, low or low-normal serum C3 levels have been associated with more severe renal disease and poorer outcomes,^5^^,^^6^ and glomerular complement deposition is frequently detected in kidneys.^7^, ^8^, ^9^ Proteomic studies have further shown that complement proteins may be present within glomeruli even when routine immunofluorescence is negative.^10^ These observations indicate that serum levels, immunofluorescence deposits, protein-level complement detection, and intrarenal complement-related gene expression represent distinct biological layers whose relationships and clinical relevance remain poorly defined.

Targeted bulk transcriptomics from diagnostic biopsies have identified complement-related genes as major correlates of kidney function and prognosis in AAV-GN,^11^ whereas spatial transcriptomics now enables compartment- and cell-type–resolved profiling of kidney tissue. Earlier intrarenal complement gene-expression work in AAV-GN^12^ further supports the relevance of tissue-level complement profiling in this setting. Whether intrarenal complement-related transcriptional programs are concordant with conventional serum and immunofluorescence markers, and whether they carry clinical information beyond these markers, remains unknown.

Here, we evaluated predefined intrarenal complement pathway-related expression scores in AAV-GN, their association with kidney severity and clinical outcomes, and their concordance with circulating C3/C4 levels and glomerular C1q/C3 deposition. We then leveraged publicly available spatial transcriptomic data sets to validate these bulk transcriptomic signatures, define their compartmental and cell-type distribution, and assess their cross-platform reproducibility.

## Methods

### Selection of Patients and Controls

As previously described,^11^^,^^13^ 2 independent cohorts of patients with AAV-GN were included in this study—the Maine-Anjou registry^14^^,^^15^ and the RENVAS registry^16^^,^^17^ (Figure 1a). Control kidney samples (*n* = 23) were included for transcriptomic comparisons (Supplementary Table S1). Detailed methods regarding patient selection, clinical data collection, outcome definitions, pathologic assessment, and ethical considerations are provided in the Supplementary Methods.

**Figure 1:**
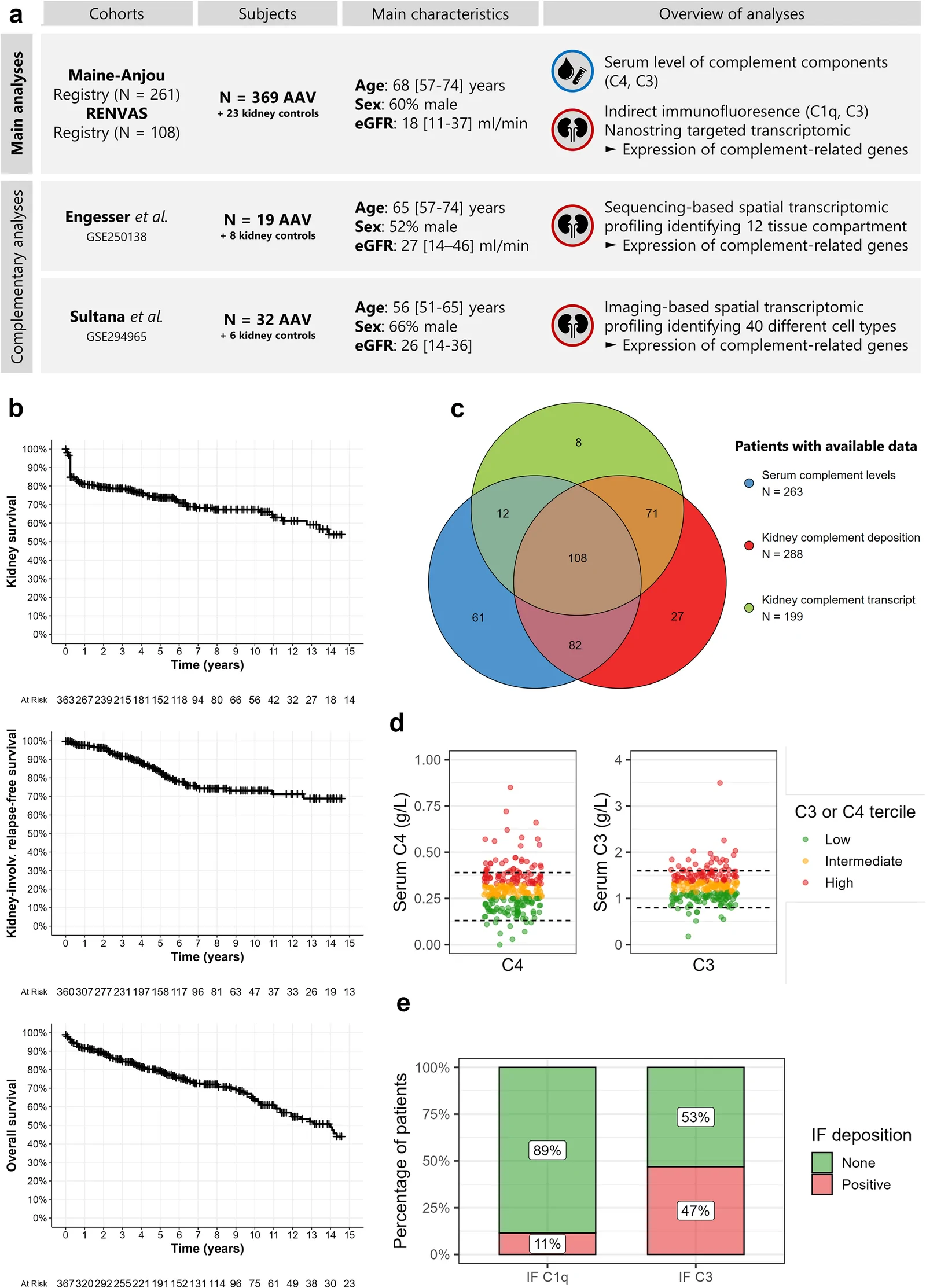
Study design, complement assessment modalities, and clinical outcomes. (a) Overview of the main AAV-GN cohort and of the 2 public spatial transcriptomic data sets analyzed in this study. (b) Kaplan–Meier estimates of kidney survival, kidney-involving relapse–free survival, and overall survival in the full AAV-GN cohort. (c) Euler diagram showing the overlap between patients with available serum complement measurements (C3 and/or C4, *n* = 263), glomerular complement deposition by immunofluorescence (C1q, *n* = 305; C3, *n* = 288), and kidney transcriptomic data. Transcriptomic control samples are not shown. (d) Distribution of serum C4 and C3 levels at diagnosis. Dashed lines indicate the lower and upper limits of the reference range. (e) Proportion of patients with detectable glomerular C1q or C3 deposition by routine immunofluorescence. Subpanels (b–e) refer to “Main analyses” mentioned in subpanel (a), carried in Maine-Anjou and RENVAS registries. AAV-GN, antineutrophil cytoplasmic antibody-associated vasculitis-glomerulonephritis; IF, immunofluorescence.

### Complement Assessment in the Main Cohort

Complement was assessed across 3 complementary layers—circulating complement levels, glomerular complement deposition, and intrarenal complement-related gene expression. Serum C3 and C4 levels were measured as part of routine clinical care before remission-induction immunosuppressive therapy. Glomerular C1q and C3 deposition were assessed by routine indirect immunofluorescence on initial diagnostic kidney biopsy and analyzed as absent versus detectable deposition. Intrarenal gene expression was assessed using targeted bulk NanoString transcriptomics using the same formalin-fixed paraffin-embedded initial diagnostic kidney biopsy specimens, as previously reported.^11^^,^^13^ The present study used the curated, normalized expression matrix for complement-focused analyses (Supplementary Methods). Predefined complement pathway–related expression scores were generated from the following selected genes: *C1QA*, *C1QB*, *C2*, and *C4A.B* for the classical pathway, and *C3*, *CFB*, *CFP*, and *CFD* for the alternative pathway. For each sample, gene-wise *z* scores were averaged to derive classical and alternative pathway expression scores, referred to as *cCP_path* and *cAP_path*, respectively. Throughout the manuscript, “pathway score” refers to these predefined transcriptomic expression scores and not to a direct biochemical measurement of complement pathway activity. *C5*, *C5AR1*, and complement regulatory genes, including *CFH*, *CD46*, *CD55*, and *CD59*, were analyzed separately.

### External Spatial Transcriptomic Data Sets

Publicly available spatial transcriptomic data sets were used to validate the complement-related transcriptional signatures identified above and to localize these signatures beyond bulk tissue expression (Figure 1a). Sequencing-based spatial transcriptomic data from Engesser *et al.*^18^ were used to assess complement-related expression across spatially annotated kidney compartments. Imaging-based spatial transcriptomic data from Sultana *et al.*^19^ were used to evaluate complement-related expression at cell-type resolution.

For both data sets, original compartment or cell-type annotations provided by the source studies were used without *de novo* reannotation. Complement pathway-related scores and individual gene-expression values were aggregated using patient-, compartment-, or cell-type-level pseudobulk approaches, as appropriate. Detailed processing steps are provided in the Supplementary Methods.

### Statistical Analysis

Continuous variables were described as median (first–third quartiles), and categorical variables as counts and percentages. Group comparisons for transcriptomic data were performed using *t* test on log2-transformed or normalized expression values, as appropriate, and effect sizes were reported as Cohen *d*. Correlations were assessed using Spearman correlation coefficient.

Survival analyses were performed using Kaplan–Meier curves, log-rank tests, and Cox proportional hazards models. Outcomes included kidney failure, kidney-involving relapse, and all-cause mortality. Multivariable Cox models were adjusted for age, sex, estimated glomerular filtration rate (eGFR) at diagnosis, and AAV phenotype. Because the objective was to evaluate associations with each outcome rather than estimate cumulative incidence in the presence of competing events, kidney failure and relapse models were interpreted as cause-specific association models; death was analyzed as a separate end point. Restricted cubic spline Cox models were used as sensitivity analyses to assess potential nonlinear associations between continuous complement markers and outcomes. Results are reported as HRs with 95% confidence intervals (CIs).

*P* values were adjusted using the Benjamini–Hochberg false-discovery rate procedure within predefined analytical families, except for Cox survival models, which were reported using nominal *P* values. Missing data were not imputed. Analyses were performed using available-case data for each complement modality and complete-case data for multivariable models. Statistical analyses were performed using R v4.0. Additional statistical methods are described in the Supplementary Methods.

## Result

### Study Population, Outcomes, and Complement Assessment Modalities

A total of 369 patients with AAV-GN were included. Baseline characteristics, treatments, and outcomes are detailed in Supplementary Table S2. Briefly, 60% of patients were male, with a predominance of microscopic polyangiitis (69%) and antimyeloperoxidase positivity (64%). Median age at diagnosis was 68 (57–74) years, median eGFR was 18 (11–37) ml/min, and median proteinuria was 1.4 (0.8–2.7) g/g. Histologically, Berden classes^20^ were distributed as follows: focal (29%), crescentic (35%), mixed (23%), and sclerotic (13%). Median interstitial fibrosis/tubular atrophy was 20%, and interstitial inflammation was graded as i1 in 54%, i2 in 28%, and i3 in 19% of patients. After a median follow-up of 60 (28–110) months, 104 of 369 (29%) patients progressed to kidney failure, 61 (17%) experienced kidney-involving relapse, and 115 (31%) died (Figure 1b).

Complement was assessed across 3 modalities—serum C3 and/or C4 in 263 (71%) patients, glomerular immunofluorescence (C1q and/or C3) in 305 (83%) and 288 (78%), respectively, and kidney transcriptomics in 199 (54%). Most patients (273/369, 74%) had ≥ 2 modalities available, and 108 (29%) had all 3 (Figure 1c). Modality-specific subsets showed broadly similar baseline characteristics to the overall cohort (Supplementary Table S2).

### Conventional Complement Markers are Associated With Severity and Outcomes

Below normal complement levels were rare (3.4% each, Figure 1d); therefore, it was analyzed using terciles. Serum C3 and C4 levels were moderately correlated (*ρ* = 0.47, *q* < 0.0001, Supplementary Figure S1B). Patients in the lowest C4 tercile were similar to those in the intermediate and high terciles, without differences in complement deposition or clinical outcomes after multivariable adjustment. In contrast, patients in the lowest C3 tercile were more likely to have myeloperoxidase-positive microscopic polyangiitis and had more severe kidney involvement at diagnosis, including lower eGFR and higher proteinuria. Low serum C3 was independently associated with kidney failure (aHR vs. intermediate tercile: 2.0 [1.1–3.7], *P* = 0.029) and death (aHR: 2.3 [1.3–4.0], *P* = 0.003), in models adjusted for age, sex, eGFR at diagnosis, and AAV phenotype (Table 1, Figure 2, Supplementary Figure S2). Restricted cubic spline analyses were consistent with higher risk at lower serum C3 values, particularly for mortality (Supplementary Figure S3).

**Table 1:** Characteristics of patients with AAV-GN according to complement levels in serum

Characteristics	Tercile of serum C4 levels					Tercile of serum C3 levels				
	*N*	Low	Intermediate	High	*q* value	*N*	Low	Intermediate	High	*q* value
		*N* = 88	*N* = 88	*N* = 87			*N* = 88	*N* = 88	*N* = 87	
Baseline characteristics										
Male gender, *n* (%)	261	48 (55%)	59 (69%)	52 (60%)	0.2	261	55 (63%)	57 (65%)	47 (55%)	0.5
Age (y)	260	70 (58–76)	71 (58, 75)	66 (54–74)	0.5	260	70 (58–75)	70 (59–74)	66 (54–74)	0.4
Disease phenotype	250				0.6	250				0.002
GPA, *n* (%)		27 (33%)	20 (25%)	25 (29%)			12 (14%)	25 (30%)	35 (42%)	
MPA, *n* (%)		56 (67%)	61 (75%)	61 (71%)			72 (86%)	57 (70%)	49 (58%)	
Organ involvement at diagnosis										
BVAS	254	17.0 (14.0–22.0)	15.0 (12.0–20.0)	16.0 (12.0–21.0)	0.5	254	16.0 (13.0–21.0)	15.0 (12.0–20.0)	18.0 (13.0–22.0)	0.2
Kidneys, *n* (%)	263	88 (100%)	88 (100%)	87 (100%)	-	263	88 (100%)	88 (100%)	87 (100%)	-
Serum creatinine (μmol/l)	261	237 (121–417)	261 (159–401)	302 (201–460)	0.5	261	318 (188–469)	289 (149–446)	228 (121–366)	0.04
eGFR (ml/min per 1.73 m^2^)	261	19 (10–41)	19 (12–36)	15 (10–29)	0.5	261	15 (9–30)	16 (10–36)	22 (12–46)	0.058
Proteinuria (g/g)	257	1.39 (0.75–2.64)	1.57 (1.03–2.80)	1.46 (0.88–3.17)	0.5	257	2.20 (1.20–3.60)	1.38 (0.84–2.49)	1.26 (0.80–2.06)	0.004
Presence of hematuria	256	79 (91%)	83 (98%)	78 (93%)	0.5	256	80 (93%)	82 (94%)	78 (94%)	0.9
Lungs (any involvement), *n* (%)	258	37 (43%)	33 (39%)	27 (31%)	0.5	258	35 (40%)	24 (28%)	38 (45%)	0.3
ENT, *n* (%)	257	36 (42%)	29 (34%)	21 (24%)	0.3	257	26 (30%)	35 (40%)	25 (30%)	0.4
Neurologic, *n* (%)	257	15 (17%)	13 (15%)	10 (12%)	0.6	257	14 (16%)	11 (13%)	13 (15%)	0.9
Biological presentation at diagnosis										
CRP (mg/l)	256	51 (21–125)	39 (16–121)	70 (26–142)	0.5	256	35 (15–83)	49 (21–121)	101 (34–198)	< 0.001
C3 level (g/l)	263	1.14 (0.97–1.32)	1.24 (1.09–1.41)	1.37 (1.20–1.54)	< 0.001	263	1.01 (0.91–1.08)	1.27 (1.20–1.32)	1.51 (1.45–1.62)	< 0.001
C4 level (g/l)	263	0.20 (0.17–0.23)	0.29 (0.27–0.31)	0.38 (0.35–0.43)	< 0.001	263	0.26 (0.18–0.30)	0.29 (0.25–0.34)	0.33 (0.28–0.38)	< 0.001
Presence of ANCA, *n* (%)	259	84 (97%)	80 (93%)	79 (92%)	0.6	259	82 (93%)	80 (92%)	81 (96%)	0.6
Type of ANCA, *n* (%)	259				0.5	259				0.012
Anti-MPO, *n* (%)		58 (67%)	60 (70%)	51 (59%)			68 (77%)	55 (63%)	46 (55%)	
Anti-PR3, *n* (%)		25 (29%)	18 (21%)	28 (33%)			12 (14%)	25 (29%)	34 (40%)	
Negative, *n* (%)		4 (4.6%)	8 (9.3%)	7 (8.1%)			8 (9.1%)	7 (8.0%)	4 (4.8%)	
Pathologic findings										
Underwent kidney biopsy	261	77 (89%)	79 (90%)	82 (95%)	0.5	261	80 (91%)	79 (91%)	79 (92%)	0.9
Normal glomeruli, %	233	30 (15–53)	27 (11–53)	29 (11–44)	0.6	233	22 (8–43)	29 (17–57)	36 (17–53)	0.13
Crescentic glomeruli, %	234	39 (18–58)	33 (19–61)	40 (23–60)	0.7	234	45 (21–64)	33 (20–54)	33 (20–57)	0.3
Sclerotic glomeruli, %	231	12 (3–26)	17 (8–34)	19 (8–33)	0.2	231	16 (9–35)	17 (5–33)	16 (5–30)	0.7
Berden histologic classification	235				0.7	235				0.4
Focal, *n* (%)		25 (32%)	22 (29%)	20 (25%)			16 (21%)	25 (32%)	26 (33%)	
Crescentic, *n* (%)		28 (36%)	25 (32%)	32 (40%)			35 (45%)	21 (27%)	29 (37%)	
Mixed, *n* (%)		17 (22%)	15 (19%)	17 (21%)			16 (21%)	19 (24%)	14 (18%)	
Sclerotic, *n* (%)		7 (9.1%)	15 (19%)	12 (15%)			11 (14%)	14 (18%)	9 (12%)	
Interstitial fibrosis/tubular atrophy (%)	187	13 (5–20)	20 (10–30)	20 (10–30)	0.5	187	20 (10–33)	13 (5–30)	20 (10–30)	0.4
Interstitial infiltrate	154				0.6	154				0.3
i1 - ≤ 25%, *n* (%)		24 (47%)	27 (56%)	36 (65%)			24 (47%)	30 (61%)	33 (61%)	
i2 - 25-50%, *n* (%)		15 (29%)	13 (27%)	11 (20%)			19 (37%)	8 (16%)	12 (22%)	
i3 - ≥ 50%, *n* (%)		12 (24%)	8 (17%)	8 (15%)			8 (16%)	11 (22%)	9 (17%)	
Immunofluorescence										
Presence of C1q deposits	201	13 (18%)	6 (9.1%)	7 (11%)	0.5	201	15 (22%)	5 (7.2%)	6 (9.4%)	0.058
Presence of C3 deposits	190	35 (54%)	28 (45%)	26 (41%)	0.5	190	37 (58%)	28 (44%)	24 (39%)	0.2
Outcomes										
Follow-up duration (mo)	263	58 (28–85)	59 (27–100)	65 (28–110)	0.6	263	55 (23–84)	58 (25–112)	68 (33–110)	0.2
Kidney-involving relapses, *n* (%)	257	11 (13%)	14 (16%)	12 (14%)	0.8	257	13 (15%)	12 (14%)	12 (14%)	0.9
Kidney failure, *n* (%)	257	17 (20%)	22 (26%)	29 (34%)	0.5	257	30 (34%)	18 (21%)	20 (23%)	0.2
Death	261	27 (31%)	34 (39%)	19 (22%)	0.3	261	36 (41%)	27 (31%)	17 (20%)	0.036

AAV-GN, antineutrophil cytoplasmic antibody-associated vasculitis with glomerulonephritis; BVAS, Birmingham Vasculitis Activity Score; CRP, C-reactive protein; ENT, ear nose throat; eGFR, estimated glomerular filtration rate; GPA: granulomatosis with polyangiitis; MPA, microscopic polyangiitis; MPO, myeloperoxidase; PR3, proteinase 3.

Patients were stratified by serum C4 and C3 terciles measured at diagnosis. *q* values correspond to Benjamini–Hochberg–adjusted *P* values for group comparisons.

**Figure 2:**
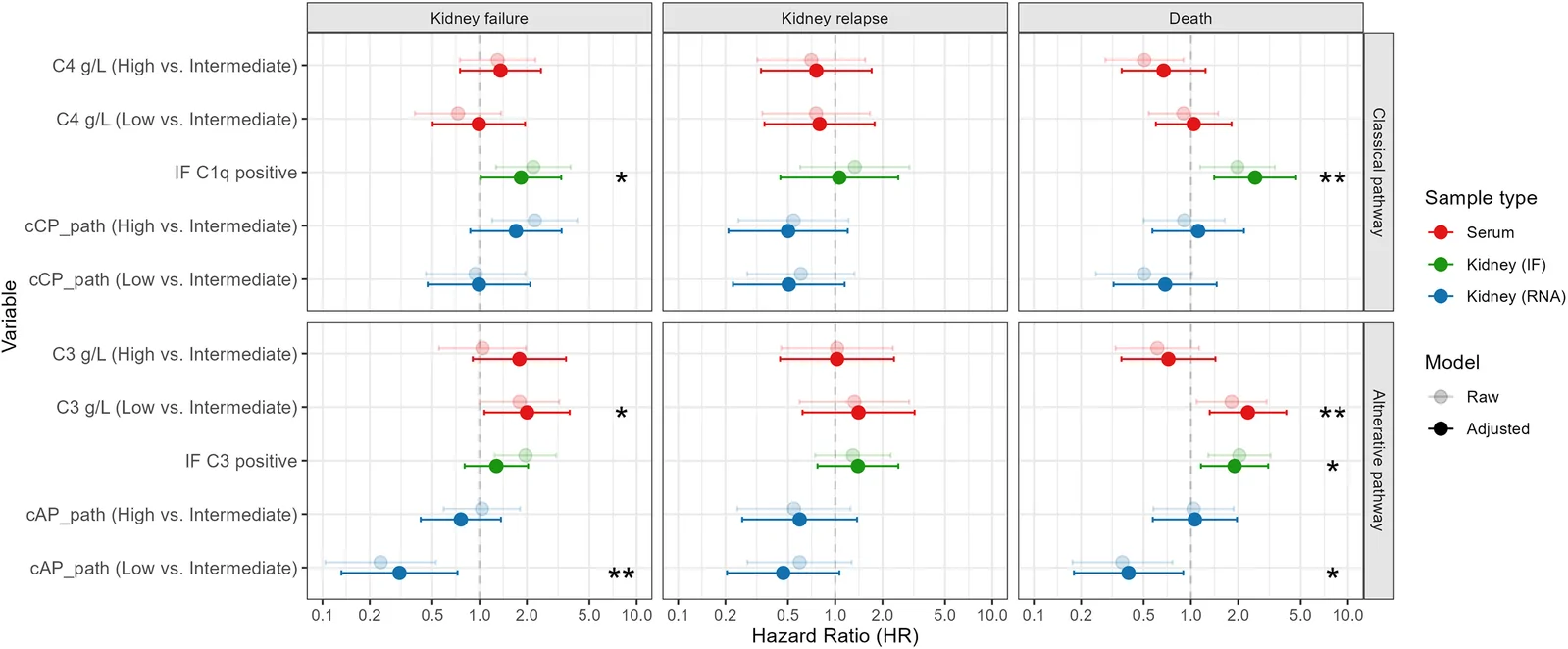
Associations between complement markers and clinical outcomes. Forest plots showing unadjusted and multivariable-adjusted hazard ratios for kidney failure, kidney-involving relapse, and death according to serum C4 and C3 terciles, glomerular C1q or C3 deposition, and kidney complement pathway-related expression score terciles. Multivariable Cox models were adjusted for age, sex, eGFR at diagnosis, and AAV phenotype. HRs are shown with 95% confidence intervals. Asterisks indicate nominal Cox model *P* values as follows: ∗*P* < 0.05, ∗∗*P* < 0.01. AAV, antineutrophil cytoplasmic antibody-associated vasculitis; eGFR, estimated glomerular filtration rate; IF, immunofluorescence.

Detectable glomerular C1q deposition was present in 11% (35/305) of patients and C3 deposition in 47% (135/288) (Figure 1e). Both were associated with more severe renal pathology. Patients who were C1q-positive had higher proteinuria, fewer normal glomeruli, greater interstitial inflammation, and lower serum C3 levels. C1q deposition was independently associated with kidney failure (aHR: 2.2 [1.3–3.8], *P* = 0.043) and death (aHR: 2.6 [1.4–4.7], *P* = 0.002). Patients who were positive for C3 had lower eGFR, fewer normal glomeruli, more crescentic glomeruli, and lower serum C3 levels. C3 deposition was independently associated with increased mortality (aHR: 1.9 [1.2–3.1], *P* = 0.011), but not with kidney failure or relapse (Table 2, Figure 2, Supplementary Figure S2).

**Table 2:** Characteristics of patients with AAV-GN according to complement deposition in kidneys

Characteristics	C1q deposition				C3 deposition			
	*N*	None	At least 1+	*q* value	*N*	None	At least 1+	*q* value
		*N* = 270 (89%)	*N* = 35 (11%)			*N* = 153 (53%)	*N* = 135 (47%)	
Baseline characteristics								
Male gender, *n* (%)	303	161 (60%)	24 (69%)	0.6	286	86 (57%)	89 (66%)	0.3
Age (yr)	305	68 (57–74)	64 (54–73)	0.5	288	67 (56–74)	68 (56–73)	0.8
Disease phenotype	291			0.6	279			0.9
GPA, *n* (%)		82 (32%)	8 (24%)			44 (30%)	40 (31%)	
MPA, *n* (%)		175 (68%)	26 (76%)			104 (70%)	91 (69%)	
Organ involvement at diagnosis								
BVAS	297	15 (12–21)	17 (14–21)	0.8	280	15 (12–21)	16 (13–20)	0.6
Kidneys, *n* (%)	305	270 (100%)	35 (100%)	-	288	153 (100%)	135 (100%)	-
Serum creatinine (μmol/l)	304	256 (146–367)	295 (186–752)	0.15	287	213 (122–342)	300 (190–429)	0.002
eGFR (ml/min per 1.73 m^2^)	304	19 (12–38)	17 (5–31)	0.3	287	23 (14–42)	15 (10–29)	0.004
Proteinuria (g/g)	296	1.23 (0.75–2.45)	2.34 (1.31–3.61)	0.014	280	1.20 (0.64–2.00)	1.64 (0.88–3.61)	0.004
Presence of hematuria	296	245 (94%)	32 (94%)	0.9	280	139 (91%)	123 (96%)	0.3
Lungs (any involvement), *n* (%)	302	91 (34%)	13 (37%)	0.9	285	47 (31%)	51 (38%)	0.5
ENT, *n* (%)	300	84 (32%)	10 (29%)	0.9	283	53 (35%)	40 (30%)	0.5
Neurologic, *n* (%)	301	39 (15%)	4 (11%)	0.9	284	25 (17%)	16 (12%)	0.4
Biological presentation at diagnosis								
CRP (mg/l)	285	56 (20–137)	37 (19–116)	0.8	268	51 (17–134)	56 (22–129)	0.6
C3 level (g/l)	201	1.27 (1.10–1.45)	1.07 (0.99–1.31)	0.047	190	1.31 (1.12–1.50)	1.22 (1.06–1.38)	0.043
C4 level (g/l)	201	0.30 (0.23–0.34)	0.26 (0.21–0.33)	0.5	190	0.31 (0.24–0.34)	0.28 (0.22–0.34)	0.5
Presence of ANCA, *n* (%)	303	252 (94%)	33 (94%)	0.9	286	146 (97%)	126 (93%)	0.4
Type of ANCA, *n* (%)	303			0.8	286			0.4
Anti-MPO, *n* (%)		172 (64%)	26 (74%)			100 (66%)	90 (67%)	
Anti-PR3, *n* (%)		76 (28%)	7 (20%)			45 (30%)	33 (24%)	
Negative, *n* (%)		20 (7.5%)	2 (5.7%)			6 (4.0%)	12 (8.9%)	
Pathologic findings								
Underwent kidney biopsy	305	270 (100%)	35 (100%)	-	288	153 (100%)	135 (100%)	-
Normal glomeruli (%)	304	32 (18–53)	12 (7–33)	0.011	287	35 (19–55)	23 (9–44)	0.008
Crescentic glomeruli (%)	303	37 (21–56)	56 (23–80)	0.15	286	36 (20–54)	44 (26–67)	0.021
Sclerotic glomeruli (%)	303	16 (6–31)	12 (7–33)	0.9	286	16 (7–31)	15 (5–33)	0.9
Berden histologic classification	304			0.082	287			0.3
Focal, *n* (%)		86 (32%)	4 (12%)			52 (34%)	30 (22%)	
Crescentic, *n* (%)		91 (34%)	19 (56%)			49 (32%)	57 (43%)	
Mixed, *n* (%)		63 (23%)	6 (18%)			36 (24%)	30 (22%)	
Sclerotic, *n* (%)		30 (11%)	5 (15%)			16 (10%)	17 (13%)	
Interstitial fibrosis/tubular atrophy (%)	239	20 (5–30)	25 (10–70)	0.065	222	20 (5–30)	20 (10–50)	0.2
Interstitial infiltrate	200			0.015	183			0.5
i1 - ≤ 25%, *n* (%)		97 (54%)	10 (45%)			48 (48%)	47 (57%)	
i2 - 25-50%, *n* (%)		42 (24%)	12 (55%)			29 (29%)	23 (28%)	
i3 - ≥ 50%, *n* (%)		39 (22%)	0 (0%)			24 (24%)	12 (15%)	
Immunofluorescence								
Presence of C1q deposits	-	-	-	-	287	6 (3.9%)	29 (21%)	<0.001
Presence of C3 deposits	287	106 (42%)	29 (83%)	< 0.001	-	-	-	-
Outcomes								
Follow-up duration (mo)	305	60 (27–111)	48 (25–97)	0.8	288	62 (35–112)	60 (27–110)	0.7
Kidney-involving relapses, *n* (%)	302	44 (16%)	7 (21%)	0.8	285	25 (17%)	26 (19%)	0.7
Kidney failure, *n* (%)	301	66 (25%)	16 (47%)	0.04	284	31 (21%)	49 (37%)	0.011
Death	305	70 (26%)	16 (46%)	0.065	288	30 (20%)	51 (38%)	0.004

AAV-GN, antineutrophil cytoplasmic antibody-associated vasculitis with glomerulonephritis; BVAS, Birmingham Vasculitis Activity Score; CRP, C-reactive protein; ENT, ear nose throat; eGFR, estimated glomerular filtration rate; GPA, granulomatosis with polyangiitis; MPA, microscopic polyangiitis; MPO, myeloperoxidase; PR3, proteinase 3.

Patients were stratified according to detectable glomerular C1q or C3 deposition by routine immunofluorescence. *q* values correspond to Benjamini–Hochberg–adjusted *P* values for group comparisons.

### Intrarenal Complement-Related Expression is Increased, Poorly Captured by Conventional Markers, and Clinically Relevant

#### Intrarenal Complement-Related Expression Programs are Increased and Linked to Inflammatory Cellular Signatures

Compared with controls, AAV-GN kidneys displayed a marked complement-related transcriptional signature within the NanoString immune panel (Figure 3a, Supplementary Figure S1A). Several complement genes were upregulated, including classical pathway components *C1QA*, *C1QB*, and *C2*, the central complement component *C3*, *C5AR1*, proximal alternative pathway-related genes *CFB* and *CFD*, and the regulatory gene *CFH*. Accordingly, predefined classical and alternative pathway–related expression scores were higher in AAV-GN kidneys than in controls (*q* < 0.0001 for both; Figure 3b).

**Figure 3:**
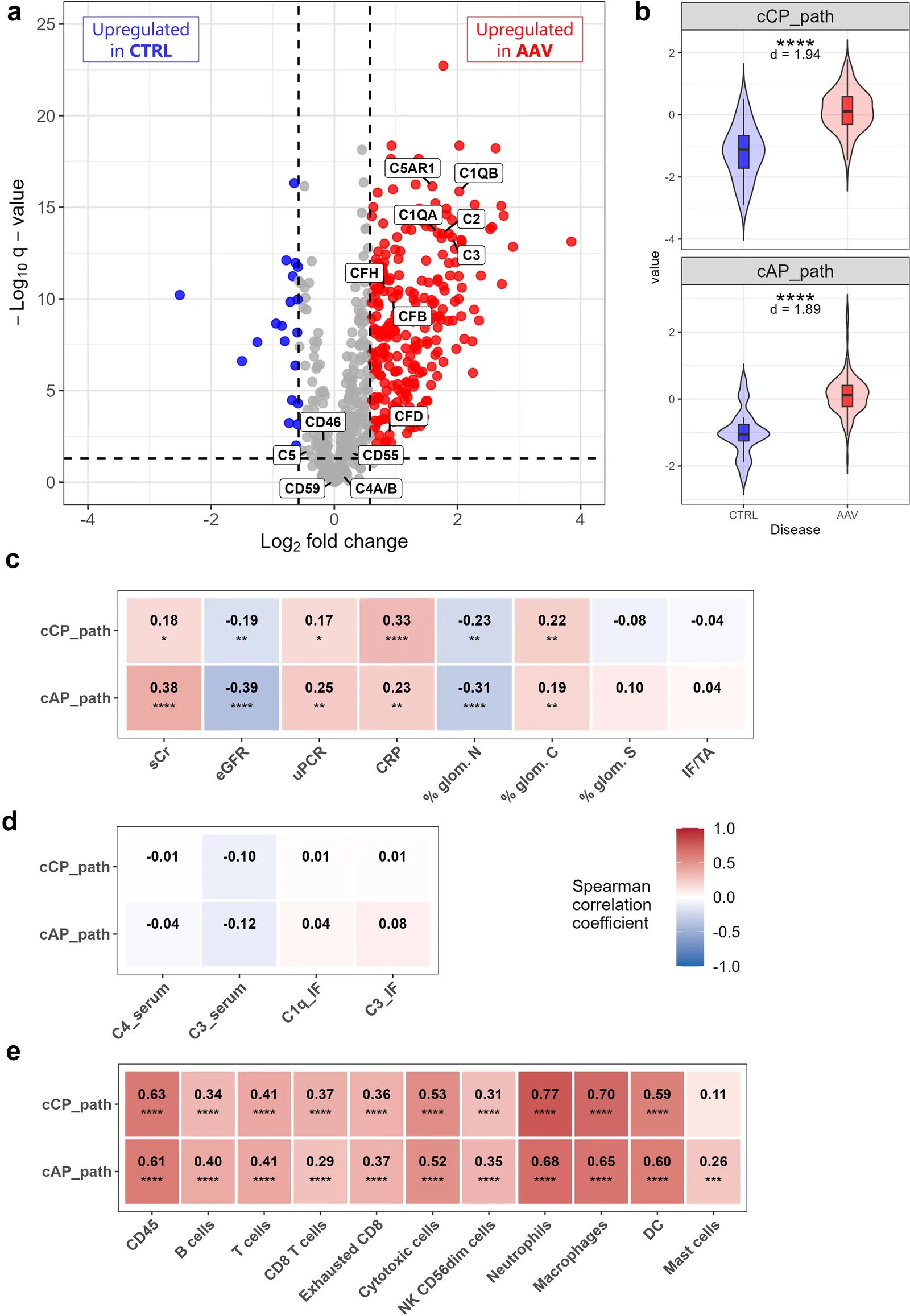
Kidney complement-related transcriptional profile in AAV-GN. (a) Volcano plot of differential gene expression across the NanoString immune panel in AAV-GN versus control kidney samples. Complement-related genes are labelled. Dashed vertical lines indicate log2 fold-change thresholds, and the dashed horizontal line indicates *q* = 0.05. (b) Distribution of predefined classical and alternative complement pathway–related expression scores in AAV-GN and control kidneys. Scores were calculated from gene-wise z scores and should be interpreted as transcriptomic expression scores. Effect sizes are shown as Cohen *d*. (c–e) Spearman correlation heatmaps showing associations between kidney complement pathway-related expression scores and clinical, biological, and histologic variables (c), conventional complement markers (d), and NanoString-derived immune cell scores (e). Clinical, biological, and histologic parameters included serum creatinine (sCr), estimated glomerular filtration rate (eGFR), urinary protein-to-creatinine ratio (uPCR), C-reactive protein (CRP), percentage of normal glomeruli (% glom. N), crescentic glomeruli (% glom. C), sclerotic glomeruli (% glom. S), and interstitial fibrosis/tubular atrophy (IF/TA). Conventional complement markers included serum C4 and C3 levels, glomerular C1q deposition, and glomerular C3 deposition. Asterisks indicate Benjamini–Hochberg–adjusted *q* values: ∗*q* < 0.05, ∗∗*q* < 0.01, ∗∗∗*q* < 0.001, ∗∗∗∗*q* < 0.0001. The color scale is shared across panels. AAV, antineutrophil cytoplasmic antibody-associated vasculitis; AAV-GN, AAV-glomerulonephritis; CTRL, control; DC, dendritic cells; NK, natural killer.

These pathway-related expression scores correlated with kidney function, proteinuria, C-reactive protein, and histologic features (Figure 3c, Supplementary Figure S1B), but showed no meaningful correlation with Birmingham Vasculitis Activity Score (Table 3). In contrast, conventional complement markers showed little concordance with intrarenal complement-related transcriptional profiles. Serum C3 and C4 levels were not correlated with *cCP_path* or *cAP_path* expression scores, and only a limited gene-level association was observed between serum C4 and kidney *C4A/B* expression. Similarly, glomerular C1q or C3 deposition was not associated with intrarenal pathway-related expression scores or with a consistent kidney complement gene-expression pattern (Figure 3d, Supplementary Figure S1B and C, Table 3).

**Table 3:** Characteristics of patients with AAV-GN according to complement pathway scores in kidneys

Characteristics	Tercile of transcriptomic classical pathway score (*cCP_path*)					Tercile of transcriptomic alternative pathway score (*cAP_path*)				
	*N*	Low	Intermediate	High	*q*-value	*N*	Low	Intermediate	High	*q* value
		*N = 67*	*N = 66*	*N = 66*			*N = 67*	*N = 66*	*N = 66*	
Baseline characteristics										
Male gender, *n* (%)	199	39 (58%)	44 (67%)	35 (53%)	0.4	199	38 (57%)	45 (68%)	35 (53%)	0.4
Age (y)	199	66 (55–72)	70 (62–75)	67 (56–75)	0.3	199	68 (57–76)	68 (58–73)	68 (57–75)	0.9
Disease phenotype	192				0.071	192				0.9
GPA, *n* (%)		18 (27%)	30 (47%)	16 (26%)			23 (35%)	22 (35%)	19 (30%)	
MPA, *n* (%)		48 (73%)	34 (53%)	46 (74%)			43 (65%)	41 (65%)	44 (70%)	
Organ involvement at diagnosis										
BVAS	196	14.0 (12.0–18.0)	17.0 (14.0–23.0)	14.5 (12.5–20.0)	0.13	196	16.0 (13.0–19.0)	15.0 (13.0–20.5)	15.0 (12.0–21.0)	0.9
Kidneys, *n* (%)	199	67 (100%)	66 (100%)	66 (100%)	-	199	67 (100%)	66 (100%)	66 (100%)	-
Serum creatinine (μmol/l)	199	243 (146–375)	256 (124–358)	313 (206–513)	0.073	199	202 (115–307)	274 (168–419)	354 (244–627)	< 0.001
eGFR (ml/min per 1.73 m^2^)	199	21 (11–41)	20 (13–40)	14 (8–23)	0.048	199	27 (15–56)	20 (11–36)	13 (7–19)	< 0.001
Proteinuria (g/g)	195	1.19 (0.61–1.89)	1.20 (0.88–2.20)	1.77 (0.89–3.63)	0.073	195	1.01 (0.56–1.70)	1.48 (1.00–2.26)	1.73 (0.80–3.78)	0.02
Presence of hematuria	195	61 (94%)	61 (94%)	62 (95%)	0.9	195	60 (92%)	61 (94%)	63 (97%)	0.9
Lungs (any involvement), *n* (%)	198	21 (31%)	31 (47%)	18 (28%)	0.11	198	24 (36%)	24 (37%)	22 (33%)	0.9
ENT, *n* (%)	198	18 (27%)	23 (35%)	13 (20%)	0.3	198	19 (28%)	18 (28%)	17 (26%)	0.9
Neurologic, *n* (%)	198	5 (7.5%)	19 (29%)	5 (7.7%)	0.004	198	10 (15%)	12 (18%)	7 (11%)	0.8
Biological presentation at diagnosis										
CRP (mg/l)	185	36 (12–69)	80 (28–151)	101 (42–170)	< 0.001	185	40 (14–124)	60 (22–129)	90 (43–168)	0.021
C3 level (g/l)	120	1.30 (1.14–1.45)	1.21 (1.09–1.38)	1.24 (1.06–1.51)	0.8	120	1.32 (1.16–1.45)	1.19 (1.06–1.46)	1.23 (1.09–1.40)	0.5
C4 level (g/l)	120	0.31 (0.23–0.36)	0.26 (0.19–0.30)	0.32 (0.24–0.37)	0.072	120	0.29 (0.24–0.34)	0.28 (0.21–0.31)	0.30 (0.21–0.37)	0.9
Presence of ANCA, *n* (%)	199	63 (94%)	63 (95%)	61 (92%)	0.9	199	63 (94%)	63 (95%)	61 (92%)	0.9
Type of ANCA, *n* (%)	199				0.4	199				0.9
Anti-MPO, *n* (%)		46 (69%)	34 (52%)	42 (64%)			40 (60%)	42 (64%)	40 (61%)	
Anti-PR3, *n* (%)		17 (25%)	26 (39%)	18 (27%)			23 (34%)	17 (26%)	21 (32%)	
Negative, *n* (%)		4 (6.0%)	6 (9.1%)	6 (9.1%)			4 (6.0%)	7 (11%)	5 (7.6%)	
Pathologic findings										
Underwent kidney biopsy	199	67 (100%)	66 (100%)	66 (100%)	-	199	67 (100%)	66 (100%)	66 (100%)	-
Normal glomeruli (%)	199	38 (21–53)	31 (19–50)	23 (8–42)	0.017	199	43 (25–55)	30 (17–44)	22 (8–35)	0.003
Crescentic glomeruli (%)	198	35 (19–50)	46 (21–60)	46 (29–67)	0.048	198	34 (17–53)	48 (29–60)	41 (24–63)	0.046
Sclerotic glomeruli (%)	198	18 (8–33)	14 (7–27)	15 (0–36)	0.7	198	14 (5–30)	17 (8–28)	18 (7–43)	0.5
Berden histologic classification	199				0.11	199				0.031
Focal, *n* (%)		22 (33%)	18 (27%)	14 (21%)			26 (39%)	16 (24%)	12 (18%)	
Crescentic, *n* (%)		18 (27%)	30 (45%)	29 (44%)			19 (28%)	30 (45%)	28 (42%)	
Mixed, *n* (%)		22 (33%)	11 (17%)	12 (18%)			17 (25%)	16 (24%)	12 (18%)	
Sclerotic, *n* (%)		5 (7.5%)	7 (11%)	11 (17%)			5 (7.5%)	4 (6.1%)	14 (21%)	
Interstitial fibrosis/tubular atrophy (%)	197	20 (10–35)	18 (5–30)	18 (6–50)	0.4	197	20 (8–30)	20 (10–30)	20 (5–50)	0.9
Interstitial infiltrate	198				0.011	198				0.025
i1 - ≤ 25%, *n* (%)		49 (73%)	30 (45%)	27 (42%)			42 (63%)	37 (57%)	27 (41%)	
i2 - 25-50%, *n* (%)		13 (19%)	23 (35%)	20 (31%)			21 (31%)	16 (25%)	19 (29%)	
i3 - ≥ 50%, *n* (%)		5 (7.5%)	13 (20%)	18 (28%)			4 (6.0%)	12 (18%)	20 (30%)	
Immunofluorescence										
Presence of C1q deposits	194	8 (12%)	6 (9.4%)	8 (12%)	0.9	194	6 (9.4%)	8 (12%)	8 (13%)	0.9
Presence of C3 deposits	179	26 (46%)	29 (49%)	28 (44%)	0.9	179	37 (62%)	26 (46%)	33 (53%)	0.5
Outcomes										
Follow-up duration (mo)	199	61 (25–101)	58 (28–97)	62 (28–118)	0.9	199	73 (32–118)	54 (28–96)	58 (24–112)	0.2
Kidney-involving relapses, *n* (%)	197	11 (16%)	15 (23%)	10 (15%)	0.6	197	12 (18%)	15 (23%)	9 (14%)	0.8
Kidney failure, *n* (%)	199	14 (21%)	15 (23%)	29 (44%)	0.029	199	8 (12%)	24 (36%)	26 (39%)	0.005
Death	199	12 (18%)	22 (33%)	22 (33%)	0.14	199	11 (16%)	22 (33%)	23 (35%)	0.1

AAV-GN, antineutrophil cytoplasmic antibody-associated vasculitis with glomerulonephritis; BVAS, Birmingham Vasculitis Activity Score; CRP, C-reactive protein; ENT, ear nose throat; eGFR, estimated glomerular filtration rate; GPA, granulomatosis with polyangiitis; MPA, microscopic polyangiitis; MPO, myeloperoxidase; PR3, proteinase 3.

Patients were stratified by terciles of predefined classical and alternative complement pathway–related expression scores derived from kidney transcriptomic data. *q* values correspond to Benjamini–Hochberg–adjusted *P* values for group comparisons.

Complement pathway-related expression scores were strongly correlated with NanoString-derived immune cell scores, particularly macrophage, neutrophil, dendritic-cell, and global leukocyte signatures (Figure 3e), indicating that bulk complement-related expression is closely linked to the inflammatory cellular architecture of AAV-GN lesions.

#### Kidney Complement Pathway–Related Expression Scores Reflect Renal Severity and are Associated With Long-Term Outcomes

Kidney complement-related genes showed strong intercorrelations (Supplementary Figure S1B). To capture pathway-level expression patterns, classical (*cCP_path*) and alternative (*cAP_path*) complement pathway-related expression scores were analyzed by terciles.

Patients with high kidney *cCP_path* score had more severe renal involvement at diagnosis, including lower eGFR, fewer normal glomeruli, and more frequent severe interstitial inflammation, compared with intermediate and low terciles. They also had higher C-reactive protein levels (Table 3). After multivariable adjustment, *cCP_path* was not independently associated with kidney failure, relapse, or death (Figure 2, Supplementary Figure S2).

High kidney *cAP_path* score was similarly associated with more severe renal disease at presentation, including lower eGFR, higher proteinuria, fewer normal glomeruli, and increased interstitial inflammation (Table 3). Low *cAP_path* expression was independently associated with lower kidney failure (aHR vs. intermediate tercile 0.3 [0.1–0.7], *P* = 0.007) and improved overall survival (aHR 0.4 [0.2–0.9], *P* = 0.03), in models adjusted for age, sex, eGFR at diagnosis, and AAV phenotype, whereas no association with kidney relapse was observed (Figure 2, Supplementary Figure S2). Restricted cubic spline analyses confirmed that the association between *cAP_path* and outcomes was not strictly monotonic and was mainly driven by a low-expression subgroup with favorable prognosis, without a clear graded increase in risk across higher *cAP_path* values (Supplementary Figure S3). Associations between individual complement gene expression and clinical outcomes are detailed in Supplementary Figure S4. Again, no association was found between *cCP_path* or *cAP_path* scores and serum C3/C4 levels or glomerular C1q/C3 deposition (Table 3).

Together, these findings indicate that intrarenal complement-related expression in AAV-GN is only partially reflected by circulating complement levels or glomerular deposition. The marked upregulation of complement-related transcripts therefore raises the question of their spatial origin within the renal parenchyma. We then used publicly available spatial transcriptomic data sets^18^^,^^19^ to assess the reproducibility of these transcriptional signatures and to localize their spatial and cellular distribution.

### Spatial Transcriptomics and Cross-Platform Meta-Analysis Support Reproducible Intrarenal Complement-Related Expression Signatures

#### Spatial Localization Across Kidney Compartments and Cell Types

Using sequencing-based spatial transcriptomics from Engesser *et al.*^18^ (Figure 1a, Supplementary Methods), patient-level pseudobulk analyses confirmed increased classical and alternative complement pathway-related expression scores in AAV-GN kidneys compared with controls, consistent with the bulk NanoString findings (Figure 4a). Spatial compartment annotation showed that these signals were not confined to overtly inflamed regions. Both *cCP_path* and *cAP_path* were increased across inflamed glomerular and tubulointerstitial compartments, but also in noninflamed glomerular, tubulointerstitial, and tubular compartments, particularly for *cAP_path* (Figure 4b–c). Individual complement gene-expression patterns across compartments are shown in Supplementary Figure S5.

**Figure 4:**
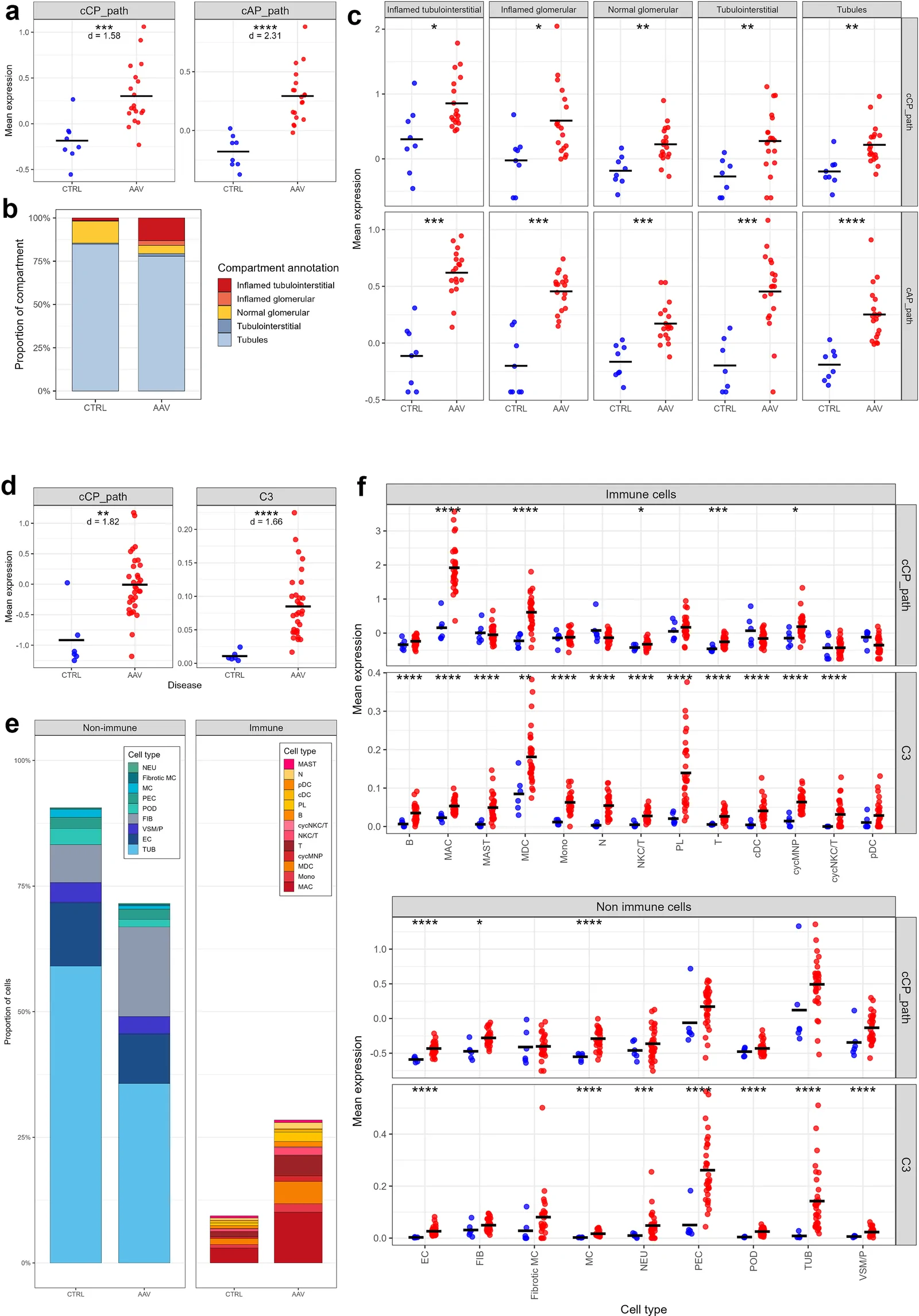
Spatial distribution of complement-related expression in AAV-GN kidneys. (a–c) Sequencing-based spatial transcriptomic analysis using public 10x Visium data from Engesser *et al.*^18^ Original spatial annotations from the source study were used. (a) Patient-level pseudobulk expression of predefined classical and alternative complement pathway–related expression scores in AAV-GN and control kidneys. (b) Relative distribution of spatial tissue compartments in AAV-GN and control kidneys. (c) Compartment-level pseudobulk expression of complement pathway–related expression scores across glomerular, tubulointerstitial, inflamed, and tubular compartments. (d–f) Imaging-based spatial transcriptomic analysis using public 10x Xenium data from Sultana *et al.*^19^ Original cell-type annotations from the source study were used. Because only C3 was available among the predefined alternative pathway genes in this data set, no alternative pathway-related score was calculated. (d) Patient-level pseudobulk expression of the classical pathway-related expression score and C3 in AAV-GN and control kidneys. (e) Relative distribution of immune and nonimmune cell populations in AAV-GN and control kidneys. (f) Cell-type–level pseudobulk expression of the classical pathway–related expression score and C3 across immune and nonimmune kidney cell populations. Asterisks indicate Benjamini–Hochberg–adjusted *q* values: ∗*q* < 0.05, ∗∗*q* < 0.01, ∗∗∗*q* < 0.001, ∗∗∗∗*q* < 0.0001. Effect sizes are shown as Cohen *d* when indicated. AAV, antineutrophil cytoplasmic antibody-associated vasculitis; cDC, conventional dendritic cell; cycNKC/T, cycling natural killer cytotoxic T cell; CTRL, control; EC, endothelial cell; FIB, fibroblast; fib. MC, fibrotic mesangial cell; MAC, macrophage; MC, mesangial cell; MDC, monocyte-derived cell; Mono, monocyte; N, neutrophil; NEU, neuronal cell; NKC/T, natural killer cytotoxic T cell; pDC, plasmacytoid dendritic cell; PEC, parietal epithelial cell; PL, plasma cell; POD, podocyte; TUB, tubular cells (grouping proximal tubule [PT], distal convoluted tubule [DCT], connecting tubule [CNT], thick ascending limb [TAL], ascending thin limb of the loop of Henle [ATL], descending thin limb of the loop of Henle [DTL], principal cell [PC], intercalated cell [IC] of the collecting duct, and papillary epithelial cells [PapE]); VSM/P, vascular smooth muscle cell/pericyte.

Imaging-based spatial transcriptomics from Sultana *et al.*^19^ further refined the cellular distribution of complement-related expression (Figure 1a, Supplementary Methods). At the patient level, AAV-GN kidneys showed increased *cCP_path* and *C3* expression compared with controls (*cAP_path* could not be calculated because *C3* was the only available gene from the predefined alternative pathway gene set in this data set; Figure 4d). Cell-type–resolved analyses showed that *cCP_path* expression was increased predominantly across immune cell populations, whereas *C3* expression was increased across both immune and nonimmune kidney cell populations (Figure 4e–f). Among immune cells, the strongest signals involved myeloid-lineage populations, including macrophages, monocyte-derived cells, and neutrophils. Among nonimmune cells, *C3* expression was particularly increased in parietal epithelial cells and tubular cells. Detailed cell-type–specific expression patterns for individual complement genes are shown in Supplementary Figures S6 and S7. In AAV-GN kidneys, *CFH* expression was predominantly observed in nonimmune kidney cell populations, whereas *C5AR1* expression was broadly distributed across immune cell types, particularly myeloid-lineage populations, with detectable expression in selected nonimmune cell populations (Supplementary Figure S7).

#### Cross-platform Reproducibility of Complement-Related Expression Signatures

To quantitatively assess the robustness of intrarenal complement-related expression we performed a cross-cohort random-effects meta-analysis of standardized effect sizes (Cohen *d*) comparing AAV-GN and control kidneys across the 3 independent transcriptomic data sets (Supplementary Methods). Detailed results are provided in Supplementary Table S3 and summarized in Figure 5.

**Figure 5:**
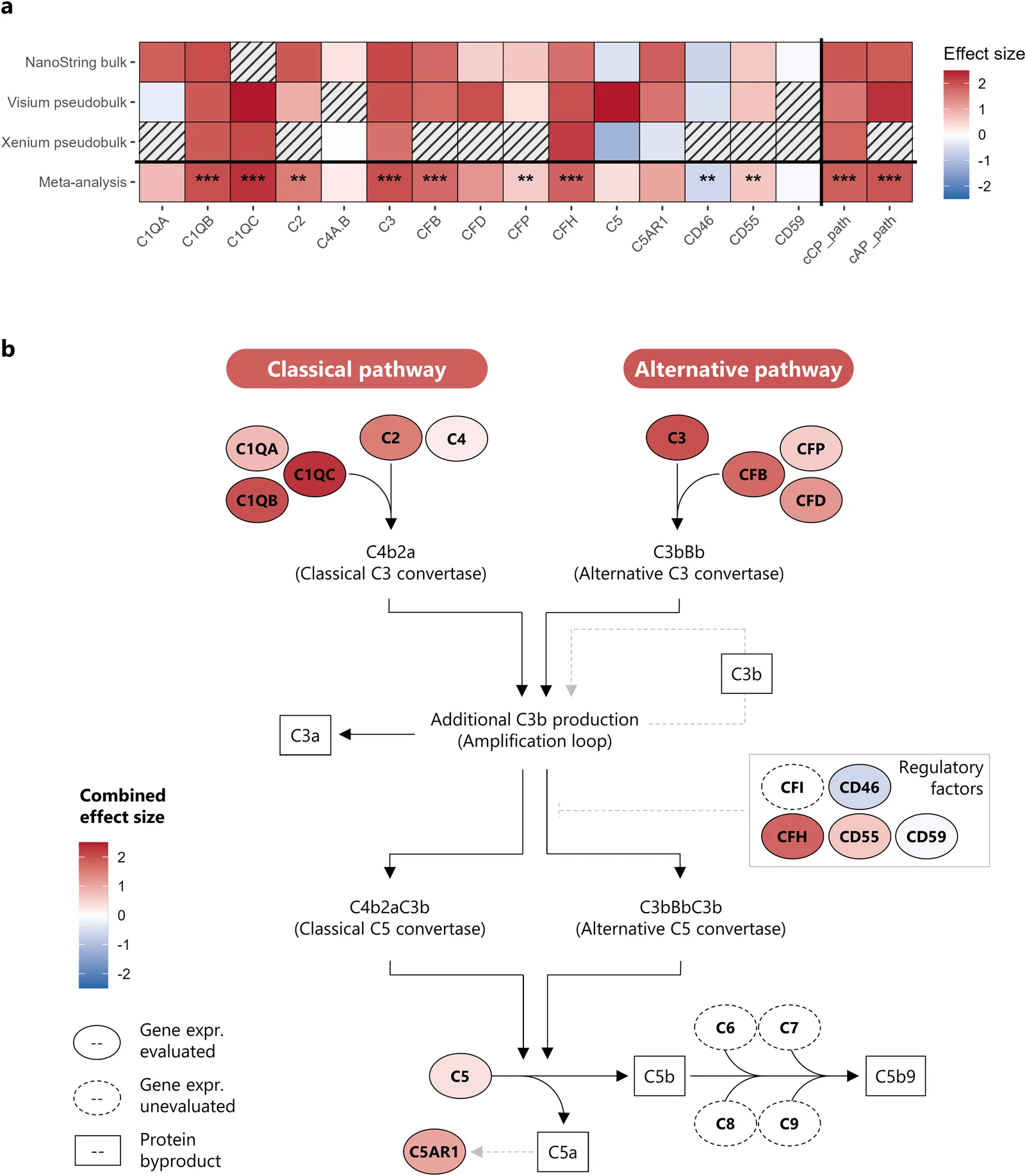
Cross-platform analysis of complement-related expression in AAV-GN. (a) Heatmap of standardized effect sizes across bulk and spatial transcriptomic data sets. Rows correspond to NanoString bulk kidney transcriptomics, Visium patient-level pseudobulk spatial transcriptomics (Engesser *et al.*^18^), Xenium patient-level pseudobulk spatial transcriptomics (Sultana *et al.*^19^), and random-effects meta-analysis. Columns correspond to complement genes and predefined pathway-related expression scores. Cell color represents Cohen *d* comparing AAV-GN and control kidneys. Hatched cells indicate genes not available in a data set. Asterisks are shown only for the random-effects meta-analysis row and indicate Benjamini–Hochberg–adjusted *q* values reported in Supplementary Table S3: ∗*q* < 0.05, ∗∗*q* < 0.01, ∗∗∗*q* < 0.001. Data set-specific statistical results are provided in the corresponding primary analyses. (b) Schematic representation of classical and alternative complement pathways annotated with random-effects meta-analysis estimates. Color intensity reflects the magnitude and direction of the pooled standardized effect size. Genes not represented in the analyzed transcriptomic data sets are outlined with dashed borders. Complement cleavage fragments and protein complexes are shown for pathway context but were not analyzed as transcriptomic features.

Several complement genes showed large and consistent effect sizes across platforms. *C1QB*, *C1QC*, *C3*, *CFB*, and *CFH* showed strong concordance (random-effects *d* ≈ 1.8–2.3; *I*^*2*^ = 0%; all *q* < 10^-8^), and both classical (*cCP_path*) and alternative (*cAP_path*) pathway-related expression scores were similarly elevated (*d* ≈ 1.9; *I*^*2*^ = 0%), supporting reproducible increased expression of both complement pathway-related signatures in AAV-GN. Among membrane-bound regulators, *CD46* was consistently decreased (*d* = −0.62; *q* = 3 × 10^-3^).

In contrast, *C5* showed marked cross-platform heterogeneity (*I*^*2*^ = 96%, *τ*^*2*^ = 4.4) with a nonsignificant pooled effect estimate, whereas *C5AR1*, *C1QA*, and *CFD* also showed substantial between-study variability (*I*^*2*^ > 85%). Given the limited number of contributing data sets per gene (*k* = 2–3), heterogeneity estimates should be interpreted cautiously.

## Discussion

In this multimodal study integrating circulating biomarkers, renal histopathology, and bulk and spatial transcriptomics in AAV-GN, we describe a layered and partially dissociated landscape of complement-related expression. AAV-GN kidneys exhibited a robust intrarenal complement-related transcriptional program involving proximal alternative pathway genes and classical pathway components, reproducibly increased across independent bulk and spatial transcriptomic data sets. This program was only weakly captured by circulating C3/C4 levels or routine glomerular immunofluorescence and was associated with kidney severity and long-term kidney and patient survival. Spatial transcriptomic data suggested contributions from both infiltrating immune cells and resident kidney compartments.

### Complement Assessment in AAV-GN is Multimodal and Partially Dissociated

Overt hypocomplementemia was uncommon, consistent with previous reports and with the historical perception of AAV-GN as a pauci-immune disease.^1^^,^^8^^,^^21^ However, low or low-normal serum C3, but not C4, was associated with more severe kidney involvement and poorer prognosis, in line with multiple independent cohorts.^5^^,^^6^^,^^22^, ^23^, ^24^, ^25^, ^26^ At the tissue level, glomerular C3 deposition was observed in nearly half of biopsies, reinforcing that complement deposition is not exceptional in AAV-GN despite its pauci-immune classification.^3^^,^^7^^,^^9^^,^^27^^,^^28^ C1q deposition was less frequent,^9^^,^^29^ but identified patients with more severe renal disease and worse outcomes, consistent with the presence of classical pathway–related transcripts in the kidney and with previous reports suggesting classical or lectin pathway involvement in selected patients.^30^, ^31^, ^32^

Importantly, correlations between circulating complement levels, glomerular complement deposition, and intrarenal complement-related gene expression were weak across modalities. These findings indicate that serum levels, routine immunofluorescence, and transcriptomic readouts capture partially distinct dimensions of complement biology in AAV-GN. Intrarenal complement-related transcripts may arise from local production by resident kidney cells, infiltrating immune cells, regulatory feedback responses, or tissue remodeling, rather than reflecting a single linear continuum from systemic consumption to tissue deposition and local gene expression.

This layered interpretation is illustrated by *CFH*, one of the most consistently increased transcripts across data sets. *CFH* encodes factor H, a major negative regulator of the alternative pathway; its upregulation may therefore reflect a local regulatory or compensatory response rather than simply stronger complement effector signaling. Similar involvement of the factor H regulatory axis has been reported in other complement-associated glomerular diseases.^33^^,^^34^ Moreover, local *CFH* transcription does not necessarily imply effective protein-level regulation, because circulating factor H is largely hepatic in origin and its biological effect depends on protein abundance, localization, surface binding, and interactions with factor H–related proteins.^35^ Thus, whether intrarenal *CFH* expression reflects effective regulation, insufficient compensation, or both cannot be resolved from transcriptomic data alone.

### Spatial Transcriptomics Suggest Multicellular Sources of Complement-Related Expression

Bulk complement pathway-related expression scores were strongly correlated with NanoString-derived immune cell signatures, especially macrophage, neutrophil, dendritic cell, and global leukocyte scores. This indicates that bulk complement-related expression is tightly coupled to the inflammatory cellular architecture of AAV-GN lesions, which is biologically expected given the central role of myeloid populations in ANCA-mediated kidney injury. However, this also highlights a key limitation of bulk transcriptomics—pathway-level expression scores cannot determine whether transcripts originate from infiltrating immune cells, resident kidney cells, or both.

Spatial transcriptomic analyses partially addressed this limitation. At the compartment level, classical and alternative pathway-related expression scores were increased not only in inflamed glomerular and tubulointerstitial regions, but also in noninflamed compartments, particularly for the alternative pathway score. At the cellular level, complement-related transcripts were detected across multiple immune and nonimmune kidney cell populations, with classical pathway expression being more prominent in immune cell populations, whereas *C3* expression was observed across both immune and resident kidney compartments, including parietal epithelial and tubular cells. These data suggest a multicellular intrarenal complement-related transcriptional response, rather than a signal exclusively driven by infiltrating leukocytes. They do not, however, prove local complement protein activation or functional pathway activity at the cellular level.

The cross-platform meta-analysis further supported the reproducibility of this transcriptional signature. *C1QB*, *C1QC*, *C3*, *CFB*, *CFH*, and the predefined classical and alternative pathway-related scores showed consistent effect sizes across independent data sets. Conversely, heterogeneity for selected transcripts, including *C5*, *C5AR1*, *C1QA*, and *CFD*, emphasizes that some complement-related signals may vary according to tissue sampling, cellular composition, platform design, spatial resolution, or analytical pipelines, warranting dedicated prospective study.

### Limitations

This study has several limitations. Its retrospective design resulted in incomplete availability of complement modalities for some patients, reflecting routine-care measurement of serum complement levels and the availability of residual diagnostic biopsy material for transcriptomic analyses. Although modality-specific subsets were broadly comparable to the overall cohort, residual selection bias cannot be excluded. Importantly, transcript abundance does not establish biochemical complement activation, and our findings should be interpreted as a descriptive characterization of complement-related transcriptional programs rather than as a direct readout of pathway activity. Indeed, we did not perform functional assays or measure circulating or urinary complement fragments (Ba/Bb, C3a, C5a, or soluble C5b-9). These assays require stringent preanalytical conditions and were not considered reliable for retrospective measurement in archived heterogeneous samples. Lastly, we also did not perform competing-risk regression or multiplicity correction across all survival contrasts; therefore, outcome analyses should be considered exploratory and hypothesis-generating.

### Translational Implications and Future Directions

Despite these caveats, the reproducibility of intrarenal complement-related expression across independent transcriptomic platforms and its association with clinical outcomes, support the relevance of tissue-level complement profiling alongside circulating biomarkers and routine immunofluorescence in AAV-GN. Given the expanding therapeutic landscape of complement modulation in nephrology,^36^ our findings highlight the potential value of intrarenal profiling to refine biological stratification and generate hypotheses for therapeutic prioritization. In particular, the consistent upregulation of proximal alternative pathway genes, classical pathway components, and regulatory transcripts such as *CFH*, suggests that future biomarker and trial designs should look beyond terminal complement readouts and also consider upstream components^37^, ^38^, ^39^, ^40^ and regulators.^41^ This may be relevant in light of emerging evidence implicating local C3–C3aR signaling in injured tubular cells as a driver of persistent inflammation and tubulointerstitial fibrosis.^42^

Prospective studies will be needed to determine whether complement-based stratification can inform trial enrichment or therapeutic decisions and ultimately improve outcomes in AAV-GN.

## Appendix

### Members of the RENVAS Registry Research Group

Franck Bourdon, Gérard Cardon, Maïté Daroux, Viviane Gnemmi, Thomas Guincestre, Maxime Hoffman, Zacharie Jomaa, Timothée Laboux, Céline Lebas, Valentine Quersin, Cyrille Vandenbussche, and Nicolas Wayolle.

### Members of the Maine-Anjou Registry Research Group

Jean-François Augusto, Céline Beauvillain, Benoit Brilland, Jean-Philippe Coindre, Marie-Christine Copin, Maud Cousin, Anne Croué, Assia Djema, Fanny Guibert, Nicolas Henry, Giorgina Barbara Piccoli, Lise-Marie Pouteau, Clément Samoreau, Emeline Vinatier, and Samuel Wacrenier.

## Disclosure

The authors declare that they have no conflicts of interest in relation to this article. All co-authors have seen and agree with the contents of the manuscript. The results presented in this paper have not been published previously in whole or part, except in abstract format.

## Supplementary Material

Supplementary File (PDF) **Supplementary Methods.****Supplementary References.****Figure S1.** Complement gene expression and relationships between complement assessment modalities. **Figure S2.** Event-free survival according to complement profile. **Figure S3.** Continuous associations between complement markers and clinical outcomes. **Figure S4.** Individual kidney complement gene expression and clinical outcomes. **Figure S5.** Compartment-level complement-related expression in the Visium spatial transcriptomic data sets. **Figure S6.** Cell-type–level complement-related expression in the Xenium spatial transcriptomic data sets. **Figure S7.** Cell-type–level complement-related expression within AAV-GN kidneys in the Xenium spatial transcriptomic data sets. **Table S1.** Characteristics of control kidney samples used for transcriptomic analyses. **Table S2.** Baseline characteristics, therapeutic management, and main outcomes in patients with AAV-GN. **Table S3.** Cross-cohort meta-analysis of complement transcriptomic effect sizes in AAV-GN. **Table S4.** STROBE checklist.

## Footnotes

Supplementary File (PDF)

**Supplementary Methods.**

**Supplementary References.**
**Figure S1.** Complement gene expression and relationships between complement assessment modalities. **Figure S2.** Event-free survival according to complement profile. **Figure S3.** Continuous associations between complement markers and clinical outcomes. **Figure S4.** Individual kidney complement gene expression and clinical outcomes. **Figure S5.** Compartment-level complement-related expression in the Visium spatial transcriptomic data sets. **Figure S6.** Cell-type–level complement-related expression in the Xenium spatial transcriptomic data sets. **Figure S7.** Cell-type–level complement-related expression within AAV-GN kidneys in the Xenium spatial transcriptomic data sets. **Table S1.** Characteristics of control kidney samples used for transcriptomic analyses. **Table S2.** Baseline characteristics, therapeutic management, and main outcomes in patients with AAV-GN. **Table S3.** Cross-cohort meta-analysis of complement transcriptomic effect sizes in AAV-GN. **Table S4.** STROBE checklist.
