# Supplementary material for: Multimodal Profiling of Complement Expression and Association With Outcomes in ANCA-Associated Vasculitis With Glomerulonephritis
Source: Kidney Int Rep. 2026 Aug 26;11(11):107056. doi: 10.1016/j.ekir.2026.107056 (PMC13657220; doi:10.1016/j.ekir.2026.107056)

Multimodal profiling of complement expression and association with outcomes in ANCA-associated vasculitis with glomerulonephritis

Benoît Brilland[1,2,3], Victor Milon[1], Cyrille Vandenbussche[4], Nathalie Merillon[2,5], Eleonore Ourouda[1], Andrea Boizard-Moracchini[6], Maëva Roy[6], Odile Blanchet[2,7], Emeline Vinatier[2,5], Céline Beauvillain[2,5], Thomas Quéméneur[4], Assia Djema[8], Nicolas Henry[9], Giorgina Barbara Piccoli[10], Samuel Wacrenier[10], Yves Delneste[2], Patrick Blanco[6,11], David Langlais[3], Viviane Gnemmi[12,13], Marie-Christine Copin[2,14], Jean-François Augusto[1,2], the RENVAS Registry Research Group, and the Maine-Anjou Registry Research Group.

1- Service de Néphrologie-Dialyse-Transplantation, CHU Angers, Angers, France
2- Univ Angers, Nantes Université, Inserm, CNRS, CRCI2NA, SFR ICAT, Angers, France
3- Department of Human Genetics, Victor Phillip Dahdaleh Institute of Genomic Medicine, McGill University, Montreal, Qc, Canada
4- Nephrology and Internal Medicine Department, Hospital of Valenciennes, Valenciennes, France
5- Laboratoire d'Immunologie et d'Allergologie, CHU d'Angers, Angers, France
6- Laboratoire d'Immunologie et Immunogénétique, FHU ACRONIM, Hôpital Pellegrin, Centre Hospitalier Universitaire de Bordeaux, France
7- Centre de Ressources Biologiques, BB-0033-00038, CHU Angers, Angers, France
8- Service de Néphrologie-Dialyse, Centre Hospitalier de Cholet, Cholet, France
9- Service de Néphrologie-Dialyse, Centre Hospitalier de Laval, Laval, France
10- Service de Néphrologie-Dialyse, Centre Hospitalier du Mans, Le Mans, France
11- CNRS-UMR 5164, ImmunoConcept, Université de Bordeaux, Bordeaux, France
12- Service d'anatomopathologie, Centre Hospitalier Universitaire de Lille, Lille, France
13- Univ. Lille, Inserm, CHU Lille, JUNIA, U1352 - BioPrev - Better aging: from inflammaging to prevention, 59000, Lille, France
14- Département de pathologie cellulaire et tissulaire, CHU Angers, Angers, France

Supplementary Material

Supplementary methods

1. Selection of patients and controls

As previously[11,13], two independent cohorts of AAV-GN patients were included in this study: the Maine-Anjou registry[14,15] and RENVAS registry[16,17]. The Maine-Anjou AAV-GN registry[14,15] gathers data on all successive AAV-GN patients diagnosed since 2000 in nephrology departments from 4 hospitals in central western France (Angers University Hospital, Le Mans, Cholet, and Laval General Hospitals). The RENVAS registry[16,17] includes data on all consecutive patients with pauci-immune glomerulonephritis diagnosed since 2000, recorded in an electronic registry held by the pathology department in northern France (gathering kidney biopsies from Lille University Hospital and Lille and Bois Bernard private Hospitals, Valenciennes, Douai, Roubaix, Béthune, Dunkerque and Boulogne-Sur-Mer General Hospitals). For the present study we considered the 2025 updates.

In both cohorts, AAV diagnosis was based on the revised 2012 Chapel Hill Consensus Conference[43]. GPA or MPA diagnosis was determined according to ACR/EULAR classification[44,45]. AAV-GN diagnosis was defined based on renal involvement (active urinary sediment with hematuria, proteinuria, and/or impaired renal function).

As previously[46–48], control subjects (n = 23) for transcriptomic kidney analysis were adult patients that underwent a kidney biopsy in the University Hospital of Angers between 2011 and 2021, with a diagnosis of minimal change disease (MCD) or benign nephroangiosclerosis (NAS), and an estimated glomerular filtration rate (eGFR) above 15 mL/min.

2. Routine data collection, pathological analysis and outcomes definition.

Collected data included baseline characteristics of patients, relevant medical history, organ involvement at AAV diagnosis and therapeutic management. Serum complement components (C3 and C4) were assessed as part of routine clinical assessment. Estimated glomerular filtration rate was calculated using the race-free Chronic Kidney Disease Epidemiology Collaboration research group equation (CKD-EPI)[49].

Most patients included in the present study underwent a kidney biopsy, analyzed in a centralized manner for each cohort (MCC and VG respectively), showing pauci-immune crescentic glomerulonephritis, confirming AAV-GN diagnosis. Berden's histopathological classification[20] was assessed to evaluate glomerular involvement. Interstitial fibrosis and tubular atrophy (IF/TA) was scored quantitatively, and interstitial infiltrate was scored semi-quantitatively (i1: ≤ 25%, i2: 25-50%, i3: ≥ 50%), as in the Banff classification[50]. C1q and C3

deposition were evaluated by indirect immunofluorescence (IIF), as part of routine pathological assessment.

Kidney failure was defined as the need for kidney replacement therapy for more than 3 months. Relapses were defined as the recurrence or renewed appearance of organ involvement attributable to AAV activity with BVAS increase (>1) and requiring an increase in steroid doses or introduction of a novel immunosuppressive drug.

3. RNA extraction from kidney biopsies.

Kidney biopsies containing at least 4 glomeruli and sufficient tissue after clinical use were selected for subsequent mRNA extraction. Ten-micrometer sections were cut from the formalin-fixed paraffin-embedded (FFPE) blocks and placed in 2 mL microcentrifuge tubes after discarding the first two sections (to avoid collecting RNA that would have been damaged by contact with air). Whole sections, including glomeruli and tubulo-interstitium were used. The number of sections per sample was dependent on the size of the FFPE tissue.

Extraction of total RNA from FFPE specimens was performed using the High Pure FFPE RNA Isolation Kit (Roche Molecular Diagnostics, Rotkreuz, Switzerland) according to the manufacturer's instructions. Extraction of RNA was either performed immediately after FFPE sectioning or sections were frozen at -20°C until the extraction was performed.

RNA purity and quantification were assessed by spectrophotometry (NanoDrop 2000; Thermo Fisher Scientific, Waltham, MA): RNA with a 260/280 ratio of 1.7–2.0 as well as a 260/230 ratio in the range of 1.8–2.3 were considered to be of acceptable quality for downstream assays). RNA quality analysis (fragmentation analysis) was performed to assess the integrity of RNA by measuring the proportion of RNA fragments greater than 200 base pairs (DV_{200}) (TapeStation; Agilent, Santa Clara, CA). The resulting data were used to calculate the corrected RNA input for each NanoString hybridization reaction following the manufacturer's instructions. Only samples with DV_{200} > 30% were considered for subsequent analyses. RNA was stored at −80 °C prior to NanoString assays. RNA was diluted in nuclease free water at a concentration of 20 ng/µL immediately before the assay, and 100 ng (5 µL) of total RNA per sample were used for quantification.

4. RNA quantification from kidney biopsies.

RNA expression in samples was measured using NanoString (nCounter Analysis System; NanoString Technologies, Seattle, WA). This method was selected over other targeted multiplex approaches because i) it requires minimal sample preparation, ii) it does not require

cDNA conversion or target amplification – both of which are major sources of variation in conventional RT-qPCR approaches, especially for FFPE samples, and iii) it has repeatedly been shown to correlate well with other microarray platforms[51–53]. This technique uses digital color-coded molecular barcode technology to measure gene expression based on the target RNA counts. It enables measurement of selected genes in a single reaction with high sensitivity and linearity across a broad dynamic range[48,52]. Samples were run according to the manufacturer's protocol on the NanoString Human Autoimmune Profiling panel with a Gene Expression Code Set profiling 770 genes (750 immunology-related human genes + 20 housekeeping genes + 14 internal controls).

Raw gene expression data were analyzed using nSolver Analysis Software v4.0 (NanoString Technologies) with the Advanced Analysis Module v2.0. The software first identifies and checks standard quality controls such as imaging, binding density, and positive controls to ensure the samples were read and reported properly. Two samples flagged for poor RNA quality were discarded before subsequent analyses.

For gene expression data, raw counts were normalized to the positive controls and then $\log_2$ transformed. To reduce technical bias, background thresholding was performed using the eight negative controls included within the panel. Genes with counts below a threshold of three standard deviations above the mean background signal were excluded from subsequent analysis. Data normalization was performed on background-thresholded samples using internal positive controls and selected housekeeping genes that were identified using the geNorm algorithm[54]. Data were normalized for hybridization and counting efficiency and kept within the range of 0.3–3 (Nanostring recommended). The positive control also normalizes all platform associated sources of variation and batch effect.

The generation, quality-control workflow, normalization strategy, and global structure of this NanoString dataset have been described in detail previously[11,13], including the study flowchart, quality-control exclusions, principal component analysis of the full 750-gene panel, differential-expression analysis, and hierarchical clustering. The present study reuses this curated and quality-controlled normalized expression matrix to perform complement-focused analyses.

5. RNA data analysis.

Differential gene expression analyses were performed using nSolver, which employs multiple multivariate linear regression models to identify significantly regulated genes (mixture negative binomial, simplified negative binomial, or log-linear model). Resulting p-values were adjusted

using the Benjamini-Hochberg (BH) (q-value) method to control the false discovery rate. Statistically significant, differentially expressed genes were defined as |log2 fold change| > 0.58 (*i.e.,* a |linear fold change| > 1.5) and q-value < 0.05 for AAV-GN patients compared to the control group.

For the present complement-focused analyses, we did not use vendor-defined NanoString pathway scores. Instead, we constructed predefined complement pathway–related expression scores using gene-wise Z-score aggregation, calculating the classical (*cCP_path*) and alternative (*cAP_path*) complement pathway scores as the mean Z-scores of genes belonging to each pathway. This approach was chosen because the analyzed gene sets were small, biologically predefined, and showed predominantly positive or neutral intra-score correlations, limiting the risk that opposing transcriptional signals would cancel each other out (**Supplementary Figure S1**). These scores should be interpreted as transcriptional expression scores, not as direct measures of biochemical complement activation.

The abundance of various cell populations was calculated using the nSolver Cell Type Profiling Module. This technique uses marker genes expressed stably and specifically in immune cell types to estimate relative abundance in sample groups measured as the average log-scale expression of their characteristic genes[55]. Note that this analysis does not provide information on the absolute number of immune cells in a sample.

6. Spatial transcriptomic analyses on public datasets.

a. Processing and analysis of sequencing-based spatial transcriptomic data from Engesser *et al.*

In the sequencing-based spatial transcriptomic dataset from Engesser et *al.*[18] (10x Visium; GSE250138), kidney biopsies from 19 AAV-GN patients and 8 controls were analyzed. Control samples corresponded to non-tumor kidney tissue obtained from tumor nephrectomy specimens. The median age of AAV-GN patients was 65 years and their median eGFR was 27 mL/min (**Figure 1a**). We did not perform de novo spatial neighborhood discovery. Instead, we used the original spatial compartment annotations provided by the authors, including their reduced five-category annotation used for visualization and downstream pseudobulk aggregation. This five-category annotation grouped the 12 original tissue compartments into normal glomerular, inflamed glomerular, tubulointerstitial, inflamed tubulointerstitial, and tubules. The broad "Tubules" category included original annotations corresponding to proximal tubules (PT), loops of Henle (LOH), connecting tubules (CNT)/principal cells (PC)/intercalated

cells (IC), PT/distal convoluted tubules (DCT), PT/thick ascending limb (TAL), DCT/CNT, CNT/PC, and mixed tubulointerstitial/vascular regions.

Among the evaluated genes, the expression of the following complement-related genes was available: *C1QA*, *C1QB*, *C1QC*, *C2*, *C3*, *C5*, *C5AR1*, *CD46*, *CD55*, *CFB*, *CFD*, *CFH*, *CFP*.

To derive complement pathway expression scores while accounting for gene-specific expression ranges, a Z-score normalization was applied at the gene level across all included capture spots (AAV-GN and selected controls). For each gene, expression values were centered and scaled using the mean and standard deviation calculated across all spots, yielding gene-wise Z-scores. Pathway scores were then computed at the spot level as the mean of Z-scores of genes belonging to the pathway of interest. Specifically, the alternative complement pathway score (*cAP_path*) was defined using *C3*, *CFB*, *CFD*, and *CFP*, and the classical complement pathway score (*cCP_path*) using *C1QA*, *C1QB*, *C1QC*, and *C2*.

These spot-level pathway scores were subsequently aggregated using a pseudobulk approach by averaging across capture spots at the patient level or within spatial compartments. Normalized expression levels of individual complement-related genes were aggregated in the same manner to generate pseudobulk expression values for each patient or compartment.

b. Processing and analysis of imaging-based spatial transcriptomic data from Sultana *et al.*

In the imaging-based spatial transcriptomic dataset from Sultana *et al.*[19] (10x Xenium; GSE294965), kidney biopsies from 32 AAV-GN patients and 6 controls were analyzed. Control samples corresponded to non-tumor kidney tissue obtained from tumor nephrectomy specimens. The median age of AAV-GN patients was 56 years and their median eGFR was 26 mL/min (**Figure 1a**). We did not perform de novo cell-type identification. Instead, we used the original cell-type annotations provided by the authors, which defined 40 cell types, including 9 tubular epithelial cell populations. For visualization and summary analyses, cell types were grouped into immune and non-immune compartments when appropriate, while the original cell-type labels were retained for cell-type–resolved analyses.

Cell types identified: cDC, conventional dendritic cell; cycNKC/T, cycling natural killer cytotoxic T cell; cycling MNP, cycling mononuclear phagocyte; EC, endothelial cell; FIB, fibroblast; fib. MC, fibrotic mesangial cell; MAC, macrophage; MC, mesangial cell; MDC, monocyte-derived cell; Mono, monocyte; N, neutrophil; NEU, neuronal cell; NKC/T, natural killer cytotoxic T cell; pDC, plasmacytoid dendritic cell; PEC, parietal epithelial cell; PL, plasma cell; podo, podocyte; TUB, tubular cells (grouping proximal tubule [PT], distal

convoluted tubule [DCT], connecting tubule [CNT], thick ascending limb [TAL], ascending thin limb of the loop of Henle [ATL], descending thin limb of the loop of Henle [DTL], principal cell [PC], intercalated cell [IC] of the collecting duct, and papillary epithelial cells [PapE]); VSM/P, vascular smooth muscle cell/pericyte.

Among the evaluated genes, the expression of the following complement-related genes was available: *C1QB*, *C1QC*, *C3*, *C4A*, *C5*, *C5AR1*, *CFH*. As above, a classical complement pathway score (*cCP_path*) was built using *C1QB*, *C1QC* and *C4A*. Because *C3* was the only gene belonging to the complement alternative pathway, no specific score was built, and this gene was selected as representative of this pathway.

As for the sequencing-based spatial dataset above, pathway scores and gene expression were subsequently aggregated using a pseudobulk approach. For the classical complement pathway, cell-level pathway scores were computed and then averaged either at the patient level or within cell types. Normalized expression levels of individual complement-related genes were aggregated in the same manner to generate pseudobulk expression values for each patient or cell type.

7. Additional statistical analysis.

STROBE checklist is provided in **Supplementary Table S4**.

a. Sample size justification.

No formal sample-size calculation was performed because this was a retrospective translational study using all available patients with AAV-GN from two regional registries and all available residual diagnostic biopsy material. Analyses were therefore considered hypothesis-generating, with emphasis on effect sizes, confidence intervals, cross-platform consistency, and FDR-adjusted results rather than isolated nominal p-values

b. Investigating non-linear association with clinical outcomes.

To assess whether associations between continuous complement markers and clinical outcomes were driven by linear trends or by non-linear patterns, restricted cubic spline Cox models were fitted for serum C3, serum C4, cAP_path, and cCP_path. Restricted cubic splines were modeled using three knots placed at the 10th, 50th, and 90th percentiles of each marker distribution, with hazard ratios referenced to the median value of each marker. Both unadjusted and multivariable-adjusted models were fitted. Adjusted models included age, sex, eGFR at diagnosis, and AAV phenotype, consistent with the primary Cox models. These analyses were

considered sensitivity analyses to complement the primary tertile-based Cox models and to evaluate whether categorization of complement markers masked non-linear associations.

c. Measurement of effect size and meta-analysis.

To assess the magnitude of the effect size between the two groups (AAV vs. CTRL), Cohen's d was calculated. Cohen's d is a standardized measure of the difference between two means, expressed in terms of pooled standard deviation. It provides an estimate of the strength of the relationship between the group and the tested variable. A Cohen's d value of 0.2 indicates a small effect, 0.5 a moderate effect, and 0.8 a large effect. This approach allows for the quantification of the clinical significance of the differences observed between groups, beyond traditional hypothesis testing. Cohen's d was computed using the *effectsize* package in R.

To assess the reproducibility of complement gene upregulation across independent transcriptomic platforms, Cohen's d were calculated for each gene and pathway score in the three independent datasets: (i) targeted bulk NanoString transcriptomics, (ii) sequencing-based spatial transcriptomics (Engesser et al.)[18], and (iii) imaging-based spatial transcriptomics (Sultana et al.)[19]. Cohen's d effect sizes were computed at the patient pseudobulk level using pooled standard deviations.

For each gene or pathway score, study-specific effect sizes were combined using inverse-variance weighted meta-analysis. The variance of Cohen's d was estimated as: $Var(d) = (n_1 + n_2)/(n_1 n_2) + d^2/[2(n_1 + n_2)]$, where n_1 and n_2 denote the control and AAV-GN sample sizes, respectively.

Random-effects models were fitted using restricted maximum likelihood (REML) estimation with the *metafor* package in R. Between-study heterogeneity was quantified using the I^2 statistic and τ^2 estimates. False discovery rate correction across the tested complement panel was performed using the Benjamini–Hochberg procedure. Random-effects estimates were preferentially interpreted to account for differences in platform technology, tissue compartment sampling, spatial resolution, and analytical pipelines across datasets.

8. Ethical considerations.

The Maine-Anjou Registry and RENVAS Registries have been declared and authorized by the "Commission National Informatique et Liberté" (CNIL, agreement number 2018-MR03-02 and number 16-898, respectively). Participants gave their written informed consent for the use of their biological material (authorization numbers DC-2014-2224 - AC-2017-2993 and 2008-642 respectively). Transcriptomic analyses were performed on kidney biopsy tissue samples

obtained during routine diagnostic/therapeutic procedures that provided enough remaining material for further research analyses.

This study was approved by the local ethics committee of the University Hospital of Angers (CE 2021-210 & 2023-064) and was conducted according to the principles of the Declaration of Helsinki and its amendments.

9. Patient and public involvement.

Patients or members of the public were not directly involved in the design, conduct, reporting, or dissemination of this retrospective study, which used previously collected registry data, archived diagnostic biopsy material, and publicly available transcriptomic datasets.

Supplementary References

S43. J.C. Jennette, R.J. Falk, P.A. Bacon, et al., 2012 Revised International Chapel Hill consensus conference nomenclature of vasculitides, Arthritis Rheum, 65, 2013, 1–11, doi:10.1002/art.37715.

S44. J.C. Robson, P.C. Grayson, C. Ponte, et al., 2022 American College of Rheumatology/European alliance of associations for rheumatology classification criteria for granulomatosis with polyangiitis, Arthritis Rheumatol, 74, 2022, 393–399, doi:10.1002/art.41986.

S45. R. Suppiah, J.C. Robson, P.C. Grayson, et al., 2022 American College of Rheumatology/European alliance of associations for rheumatology classification criteria for microscopic polyangiitis, Arthritis Rheumatol, 74, 2022, 400–406, doi:10.1002/art.41983.

S46. J.B. Hodgin, A.C. Borczuk, S.H. Nasr, et al., A molecular profile of focal segmental glomerulosclerosis from formalin-fixed, paraffin-embedded tissue, Am J Pathol, 177, 2010, 1674–1686, doi:10.2353/
ajpath.2010.090746.

S47. C.C. Berthier, H. Zhang, M. Schin, et al., Enhanced expression of Janus Kinase-Signal transducer and activator of transcription pathway members in human diabetic nephropathy, Diabetes, 58, 2009, 469–477, doi:10.2337/db08-1328.

S48. J.M. Mejia-Vilet, S.V. Parikh, H. Song, et al., Immune gene expression in kidney biopsies of lupus nephritis patients at diagnosis and at renal flare, Nephrol Dial Transplant, 34, 2019, 1197–1206, doi:10.1093/ndt/gfy125.

S49. L.A. Inker, N.D. Eneanya, J. Coresh, et al., New creatinine- and cystatin C–based equations to estimate GFR without race, N Engl J Med, 385, 2021, 1737–1749, doi:10.1056/NEJMoa2102953.

S50. M. Mengel, A. Loupy, M. Haas, et al., Banff 2019 Meeting Report: molecular diagnostics in solid organ transplantation–Consensus for the Banff Human Organ Transplant (B-HOT) gene panel and open source multicenter validation, Am J Transplant, 20, 2020, 2305–2317, doi:10.1111/ajt.16059.

S51. P.P. Reis, L. Waldron, R.S. Goswami, et al., mRNA transcript quantification in archival samples using multiplexed, color-coded probes, BMC Biotechnol, 11, 2011, 46, doi:10.1186/1472-6750-11-46.

S52. H.F. Tsang, V.W. Xue, S.P. Koh, Y.M. Chiu, L.P.W. Ng and S.C.C. Wong, NanoString, a novel digital color-coded barcode technology: current and future applications in molecular diagnostics, Expert Rev Mol Diagn, 17, 2017, 95–103, doi:10.1080/14737159.2017.1268533.

S53. S.D. Prokopec, J.D. Watson, D.M. Waggott, et al., Systematic evaluation of medium-throughput mRNA abundance platforms, RNA, 19, 2013, 51–62, doi:10.1261/rna.034710.112.

S54. J. Vandesompele, K. De Preter, F. Pattyn, et al., Accurate normalization of real-time quantitative RT-PCR data by geometric averaging of multiple internal control genes, Genome Biol, 3, 2002, RESEARCH0034, doi:10.1186/gb-2002-3-7-research0034.

S55. P. Danaher, S. Warren, L. Dennis, et al., Gene expression markers of Tumor Infiltrating leukocytes, J Immunother Cancer, 5, 2017, 18, doi:10.1186/s40425-017-0215-8.

Supplementary Figures Legends

Supplementary Figure S1. Complement gene expression and relationships between complement assessment modalities.

(a) Differential expression of complement-related genes in AAV-GN versus control kidney samples, assessed by targeted NanoString transcriptomics. Genes are grouped by complement pathway annotation. Effect sizes are shown as Cohen's d.

(b) Correlation matrix showing Spearman correlations between serum complement levels, glomerular complement deposition, kidney complement gene expression, complement pathway–related expression scores, and clinical, biological, or histological variables. Boxed areas highlight relationships between serum complement levels and kidney complement-related expression (Box 1), between glomerular complement deposition and kidney complement-related expression (Box 2), and within the predefined classical (*cCP_path*) and alternative pathway gene sets (*cAP_path*) (Box 3 and 4).

(c) Scatter plot showing the association between serum C4 level and kidney C4A/B expression. Asterisks indicate Benjamini–Hochberg–adjusted q-values: * $q < 0.05$, ** $q < 0.01$, *** $q < 0.001$, **** $q < 0.0001$.

Supplementary Figure S2. Event-free survival according to complement profile.

Kaplan–Meier curves showing kidney survival **(a)**, kidney-involving relapse–free survival **(b)**, and overall survival **(c)** according to serum complement levels, glomerular complement deposition, and kidney complement pathway–related expression scores. p-values, when displayed, correspond to nominal log-rank tests.

Supplementary Figure S3. Continuous associations between complement markers and clinical outcomes.

Restricted cubic spline Cox models showing associations between serum C4, serum C3, the classical pathway–related expression score (*cCP_path*), the alternative pathway–related expression score (*cAP_path*), and clinical outcomes. Hazard ratios are referenced to the median value of each marker and displayed on a log scale. Blue curves represent multivariable-adjusted models, with shaded 95% confidence intervals. Adjusted models included age, sex, eGFR at diagnosis, and AAV phenotype. The horizontal dashed line indicates HR = 1.

Supplementary Figure S4. Individual kidney complement gene expression and clinical outcomes.

Forest plots showing unadjusted and multivariable-adjusted hazard ratios for kidney failure, kidney-involving relapse, and death according to kidney complement gene-expression terciles. Multivariable Cox models were adjusted for age, sex, eGFR at diagnosis, and AAV phenotype. Hazard ratios are shown with 95% confidence intervals. Asterisks indicate nominal Cox model p-values: * $p < 0.05$, ** $p < 0.01$, *** $p < 0.001$, **** $p < 0.0001$.

Supplementary Figure S5. Compartment-level complement-related expression in the Visium spatial transcriptomic data sets.

(a) Patient-level pseudobulk expression of individual complement genes in AAV-GN and control kidneys.

(b) Compartment-level pseudobulk expression of individual complement genes across spatially annotated kidney compartments.

Original annotations from Engesser *et al.* [18]. were used without de novo reannotation. Expression values represent mean log-normalized expression aggregated at the patient level, either globally or within each tissue compartment. Asterisks indicate Benjamini–Hochberg–adjusted q-values: * $q < 0.05$, ** $q < 0.01$, *** $q < 0.001$, **** $q < 0.0001$.

Supplementary Figure S6. Cell-type–level complement-related expression in the Xenium spatial transcriptomic data sets.

(a) Patient-level pseudobulk expression of complement-related genes in AAV-GN and control kidneys.

(b) Cell-type–level pseudobulk expression of complement-related genes across immune and non-immune kidney cell populations.

Original annotations from Sultana *et al.*[19]. were used without de novo cell-type reannotation. Expression values represent mean log-normalized expression aggregated at the patient level, either globally or within each cell type. Asterisks indicate Benjamini–Hochberg–adjusted q-values: * $q < 0.05$, ** $q < 0.01$, *** $q < 0.001$, **** $q < 0.0001$.

Cell-type abbreviations: B, B cell; cDC, conventional dendritic cell; cycMNP, cycling mononuclear phagocyte; cycNKC/T, cycling natural killer/cytotoxic T cell; EC, endothelial cell; FIB, fibroblast; fibrotic MC, fibrotic mesangial cell; MAC, macrophage; MC, mesangial cell; MDC, monocyte-derived cell; Mono, monocyte; N, neutrophil; NEU, neuronal cell; NKC/T, natural killer/cytotoxic T cell; pDC, plasmacytoid dendritic cell; PEC, parietal

epithelial cell; PL, plasma cell; POD, podocyte; T, T cell; TUB, tubular cells; VSM/P, vascular smooth muscle cell/pericyte.

Supplementary Figure S7. Cell-type–level complement-related expression within AAV-GN kidneys in the Xenium spatial transcriptomic data sets.

Cell-type–level pseudobulk expression of complement-related genes across immune and non-immune kidney cell populations among AAV-GN patients only. This analysis describes the distribution of complement-related transcripts within AAV-GN kidneys and does not include between-group testing. Original cell-type annotations from Sultana *et al.*[19]. were used without de novo reannotation. Expression values represent mean log-normalized expression aggregated at the patient level within each cell type. Cell-type abbreviations are provided in **Supplementary Figure S6**.

Supplementary Tables legends

Supplementary Table S1. Characteristics of control kidney samples used for transcriptomic analyses.

Clinical, biological, and pathological characteristics of control subjects included in the kidney transcriptomic comparisons.

CRP, C-reactive protein; eGFR, estimated glomerular filtration rate; MCD, minimal change disease; NAS, nephroangiosclerosis.

Supplementary Table S2. Baseline characteristics, therapeutic management and main outcomes of AAV-GN patients.

Characteristics are shown for the full cohort and for modality-specific subsets according to available serum complement measurements, glomerular immunofluorescence data, and kidney transcriptomic data.

AAV-GN, ANCA-associated Vasculitis with Glomerulonephritis; BVAS, Birmingham Vasculitis Activity Score; CRP, C-reactive protein; ENT, Ear Nose Throat; eGFR, estimated Glomerular Filtration Rate; GPA: granulomatosis with polyangiitis; MPA, microscopic polyangiitis; MPO, Myeloperoxidase; PR3, Proteinase 3.

Supplementary Table S3. Cross-cohort meta-analysis of complement transcriptomic effect sizes in AAV-GN.

Random-effects meta-analysis of standardized effect sizes comparing AAV-GN and control kidneys across available transcriptomic datasets: NanoString bulk kidney transcriptomics, Visium patient-level pseudobulk spatial transcriptomics, and Xenium patient-level pseudobulk spatial transcriptomics. Study-specific Cohen's d estimates were combined using inverse-variance weighting with restricted maximum likelihood estimation. The number of contributing datasets is indicated for each gene or pathway-related expression score. RE_low and RE_high correspond to the 95% confidence interval bounds of the random-effects estimate. I^2 and τ^2 describe between-dataset heterogeneity. p_random indicates the nominal p-value for the pooled random-effects estimate, and q_random indicates the Benjamini–Hochberg–adjusted q-value across the tested complement panel. Pathway-related expression scores (*cAP_path* and *cCP_path*) were derived from gene-wise Z-score aggregation as described in the **Supplementary Methods**. Heterogeneity estimates should be interpreted cautiously when based on a limited number of datasets.

Supplementary Table S4. STROBE checklist.

Supplementary Table S1. Characteristics of control kidney samples used for transcriptomic analyses.

	**Control subjects N = 23**
Age - years	57 (46, 68)
Sex - male, n (%)	10 (43%)
BMI - kg/m²	27.8 (23.6, 29.1)
Pathological diagnosis	
MCD	12 (52%)
NAS	11 (48%)
Serum creatinine - µmol/L	100 (66, 154)
eGFR - mL/min	59 (36, 98)
Proteinuria - g/g	1.09 (0.36, 4.29)
CRP - mg/L	4 (3, 5)

Supplementary Table S2. Baseline characteristics, therapeutic management and main outcomes of AAV-GN patients.

Characteristics	N	All cohort *N = 369*	Serum (C3 and C4) *N = 263*	IF (C1q and C3) *N = 288*	RNA and pathways *N = 199*
**Baseline characteristics**					
Male gender, n (%)	367	221 (60%)	159 (61%)	175 (61%)	118 (59%)
Age - years	366	68 (57, 74)	69 (57, 74)	67 (56, 74)	68 (57, 75)
**Disease phenotype**	352				
GPA, n (%)		109 (31%)	72 (29%)	84 (30%)	64 (33%)
MPA, n (%)		243 (69%)	178 (71%)	195 (70%)	128 (67%)
**Organ involvement at diagnosis**					
BVAS	359	16 (12, 21)	16.0 (12.0, 21.0)	16 (12, 21)	15.0 (13.0, 20.0)
Kidneys, n (%)	369	369 (100%)	263 (100%)	288 (100%)	199 (100%)
Serum creatinine - µmol/L	366	271 (151, 406)	273 (151, 424)	256 (149, 375)	274 (153, 419)
eGFR - ml/min/1,73m²	366	18 (11, 37)	17 (11, 37)	19 (12, 38)	17 (10, 36)
Proteinuria - g/g	357	1.39 (0.80, 2.68)	1.49 (0.87, 2.71)	1.36 (0.80, 2.62)	1.38 (0.80, 2.50)
Presence of haematuria	356	333 (94%)	240 (94%)	262 (94%)	184 (94%)
Lungs (any involvement), n (%)	364	131 (36%)	97 (38%)	98 (34%)	70 (35%)
ENT, n (%)	362	113 (31%)	86 (33%)	93 (33%)	54 (27%)
Neurological, n (%)	363	55 (15%)	38 (15%)	41 (14%)	29 (15%)
**Biological presentation at diagnosis**					
CRP - mg/L	345	56 (21, 134)	55 (21, 133)	53 (20, 134)	60 (22, 142)
C3 level - g/L	263	1.26 (1.08, 1.45)	1.26 (1.08, 1.45)	1.25 (1.08, 1.45)	1.27 (1.09, 1.45)
C4 level - g/L	263	0.29 (0.23, 0.35)	0.29 (0.23, 0.35)	0.29 (0.23, 0.34)	0.29 (0.22, 0.35)
Presence of ANCA, n (%)	365	343 (94%)	243 (94%)	272 (95%)	187 (94%)
Type of ANCA, n (%)	365				
anti-MPO, n (%)		234 (64%)	169 (65%)	190 (66%)	122 (61%)
anti-PR3, n (%)		105 (29%)	71 (27%)	78 (27%)	61 (31%)
negative, n (%)		26 (7.1%)	19 (7.3%)	18 (6.3%)	16 (8.0%)
**Pathological findings**					
Underwent kidney biopsy	367	344 (94%)	238 (91%)		
Normal glomeruli - %	339	30 (13, 53)	29 (11, 52)	30 (13, 53)	30 (14, 50)
Crescentic glomeruli - %	340	37 (21, 59)	36 (20, 60)	38 (21, 60)	42 (22, 59)
Sclerotic glomeruli - %	337	16 (7, 33)	17 (7, 33)	16 (6, 33)	17 (7, 33)
Berden histological classification	341				
Focal, n (%)		98 (29%)	67 (29%)	82 (29%)	54 (27%)
Crescentic, n (%)		120 (35%)	85 (36%)	106 (37%)	77 (39%)
Mixed, n (%)		80 (23%)	49 (21%)	66 (23%)	45 (23%)
Sclerotic, n (%)		43 (13%)	34 (14%)	33 (11%)	23 (12%)
Interstitial fibrosis/tubular atrophy (%)	272	20 (8, 30)	20 (8, 30)	20 (8, 30)	20 (8, 30)
Interstitial infiltrate	232				
i1 - ≤ 25%, n (%)		125 (54%)	87 (56%)	95 (52%)	106 (54%)
i2 - 25-50%, n (%)		64 (28%)	39 (25%)	52 (28%)	56 (28%)
i3 - ≥ 50%, n (%)		43 (19%)	28 (18%)	36 (20%)	36 (18%)
Immunofluorescence					
Presence of C3 deposits	288	135 (47%)	89 (47%)	135 (47%)	83 (46%)
Presence of C1q deposits	305	35 (11%)	26 (13%)	35 (12%)	22 (11%)
**Therapeutic management**					
Induction remission therapy					
Plasma exchange, n (%)	364	83 (23%)	61 (24%)	61 (21%)	53 (27%)
Intravenous corticoids, n (%)	359	280 (78%)	199 (78%)	224 (79%)	161 (81%)
Oral corticosteroid, n (%)	354	350 (99%)	248 (98%)	278 (99%)	197 (99%)
Cyclophosphamide, n (%)	359	266 (74%)	183 (71%)	207 (74%)	153 (77%)
Rituximab, n (%)	356	95 (27%)	77 (30%)	80 (29%)	51 (26%)
Maintenance therapy					
Rituximab, n (%)	349	185 (53%)	142 (56%)	152 (56%)	110 (58%)
Azathioprine, n (%)	328	131 (40%)	79 (34%)	108 (43%)	65 (36%)
**Outcomes**					
Follow-up duration - months	369	60 (28, 110)	60 (28, 100)	60 (28, 112)	60 (28, 105)
Kidney-involving relapses, n (%)	361	61 (17%)	37 (14%)	51 (18%)	36 (18%)
Kidney failure, n (%)	363	104 (29%)	68 (26%)	80 (28%)	58 (29%)
Death	367	115 (31%)	80 (31%)	81 (28%)	56 (28%)

Supplementary Table S3. Cross-cohort meta-analysis of complement transcriptomic effect sizes in AAV-GN.

Pathway	Gene (or pathway score)	N. of datasets	d_random	RE_low	RE_high	I² (%)	τ²	p_random	q_random	p_heterogeneity
Classical	**cCP_path**	3	1.86	1.48	2.24	0	0.0	1.98E-21	1.12E-20	0.799
	C1QA	2	0.81	-1.33	2.95	95	2.3	0.457	0.518	7.10E-06
	C1QB	3	2.02	1.63	2.41	0	0.0	2.92E-24	3.19E-23	0.932
	C1QC	2	2.31	1.58	3.04	0	0.0	5.08E-10	1.23E-09	0.492
	C2	2	1.51	0.56	2.46	74	0.4	0.002	0.003	0.051
	C4A.B	2	0.25	-0.14	0.63	0	0.0	0.214	0.260	0.476
Alternative	**cAP_path**	2	1.96	1.53	2.38	0	0.0	1.53E-19	5.19E-19	0.465
	C3	3	2.01	1.62	2.40	0	0.0	3.75E-24	3.19E-23	0.708
	CFB	2	1.75	1.34	2.17	0	0.0	1.29E-16	3.65E-16	0.957
	CFD	2	1.23	-0.20	2.66	86	0.9	0.091	0.129	0.008
	CFP	2	0.59	0.21	0.98	0	0.0	0.003	0.004	0.538
Regulators	CFH	3	1.80	1.42	2.19	0	0.0	3.76E-20	1.60E-19	0.558
	CD46	2	-0.62	-1.00	-0.23	0	0.0	0.002	0.003	0.738
	CD55	2	0.65	0.26	1.03	0	0.0	0.001	0.002	0.872
	CD59	1	-0.09	-0.52	0.34	-	-	0.690	0.733	-
C5/C5AR1 axis	C5	3	0.39	-2.03	2.81	96	4.4	0.752	0.752	2.99E-08
	C5AR1	3	1.05	-0.34	2.45	90	1.4	0.139	0.182	4.23E-05

Supplementary Table S4. STROBE checklist.

STROBE Statement—checklist of items that should be included in reports of observational studies

	Item No.	Recommendation	Page No.	Relevant text from manuscript
**Title and abstract**	1	(*a*) Indicate the study's design with a commonly used term in the title or the abstract	1	
		(*b*) Provide in the abstract an informative and balanced summary of what was done and what was found	3	
**Introduction**				
Background/rationale	2	Explain the scientific background and rationale for the investigation being reported	5	
Objectives	3	State specific objectives, including any prespecified hypotheses	5	
**Methods**				
Study design	4	Present key elements of study design early in the paper	6-7	
Setting	5	Describe the setting, locations, and relevant dates, including periods of recruitment, exposure, follow-up, and data collection	6 (section II.1) And sup methods	
Participants	6	(*a*) *Case-control study*—Give the eligibility criteria, and the sources and methods of case ascertainment and control selection. Give the rationale for the choice of cases and controls	6 (section II.1) And sup methods	
		(*b*) *Case-control study*—For matched studies, give matching criteria and the number of controls per case	6 (section II.1) And sup methods	
Variables	7	Clearly define all outcomes, exposures, predictors, potential confounders, and effect modifiers. Give diagnostic criteria, if applicable	6 (sections II.1) And sup methods	
Data sources/ measurement	8*	For each variable of interest, give sources of data and details of methods of assessment (measurement). Describe comparability of assessment methods if there is more than one group	6 (sections II.1) And sup methods	
Bias	9	Describe any efforts to address potential sources of bias	7 (section II.4)	Adjusted survival analyses
Study size	10	Explain how the study size was arrived at	8 (section III.2) And sup methods 7a	Figure 1c

Continued on next page

Quantitative variables	11	Explain how quantitative variables were handled in the analyses. If applicable, describe which groupings were chosen and why	7 (section II.4)
Statistical methods	12	(*a*) Describe all statistical methods, including those used to control for confounding	7 (section II.4)<br>And sup methods
		(*b*) Describe any methods used to examine subgroups and interactions	NA
		(*c*) Explain how missing data were addressed	7 (section II.4)
		(*d*) *Case-control study*—If applicable, explain how matching of cases and controls was addressed	NA
		(*e*) Describe any sensitivity analyses	NA
**Results**			
Participants	13*	(a) Report numbers of individuals at each stage of study—eg numbers potentially eligible, examined for eligibility, confirmed eligible, included in the study, completing follow-up, and analysed	Figure 1c<br>Supplementary Table 1
		(b) Give reasons for non-participation at each stage	NA
		(c) Consider use of a flow diagram	NA
Descriptive data	14*	(a) Give characteristics of study participants (eg demographic, clinical, social) and information on exposures and potential confounders	8 (section III.1)
		(b) Indicate number of participants with missing data for each variable of interest	Done
Outcome data	15*	*Case-control study*—Report numbers in each exposure category, or summary measures of exposure	Done
Main results	16	(*a*) Give unadjusted estimates and, if applicable, confounder-adjusted estimates and their precision (eg, 95% confidence interval). Make clear which confounders were adjusted for and why they were included	Done
		(*b*) Report category boundaries when continuous variables were categorized	NA

Continued on next page

Other analyses	17	Report other analyses done—eg analyses of subgroups and interactions, and sensitivity analyses	NA
**Discussion**			
Key results	18	Summarise key results with reference to study objectives	13-16
Limitations	19	Discuss limitations of the study, taking into account sources of potential bias or imprecision. Discuss both direction and magnitude of any potential bias	15
Interpretation	20	Give a cautious overall interpretation of results considering objectives, limitations, multiplicity of analyses, results from similar studies, and other relevant evidence	13-16
Generalisability	21	Discuss the generalisability (external validity) of the study results	15-16
**Other information**			
Funding	22	Give the source of funding and the role of the funders for the present study and, if applicable, for the original study on which the present article is based	Done

*Give information separately for cases and controls in case-control studies and, if applicable, for exposed and unexposed groups in cohort and cross-sectional studies.

Note: An Explanation and Elaboration article discusses each checklist item and gives methodological background and published examples of transparent reporting. The STROBE checklist is best used in conjunction with this article (freely available on the Web sites of PLoS Medicine at http://www.plosmedicine.org/, Annals of Internal Medicine at http://www.annals.org/, and Epidemiology at http://www.epidem.com/). Information on the STROBE Initiative is available at www.strobe-statement.org.

Supplementary Figure S1

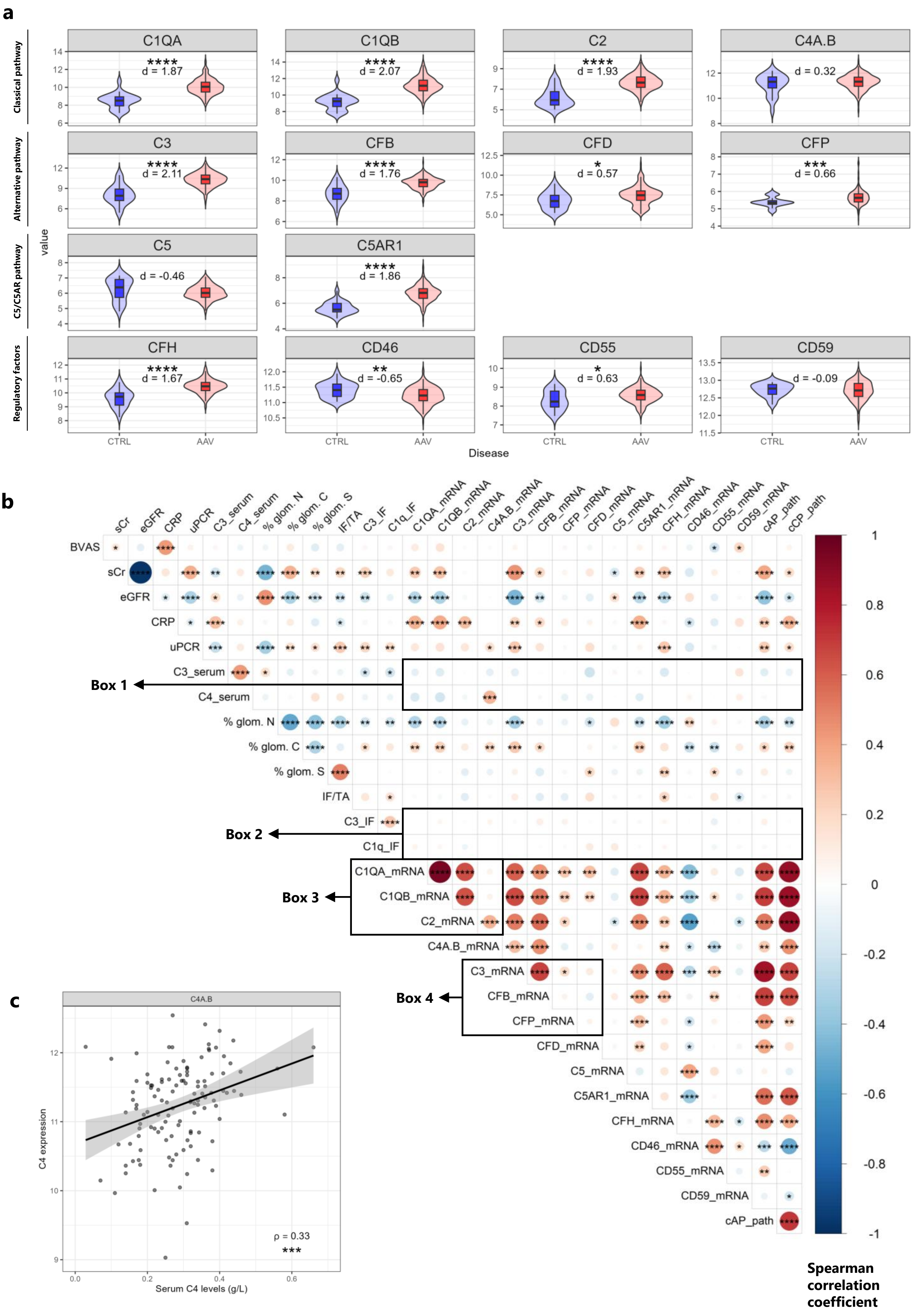

Supplementary Figure S2

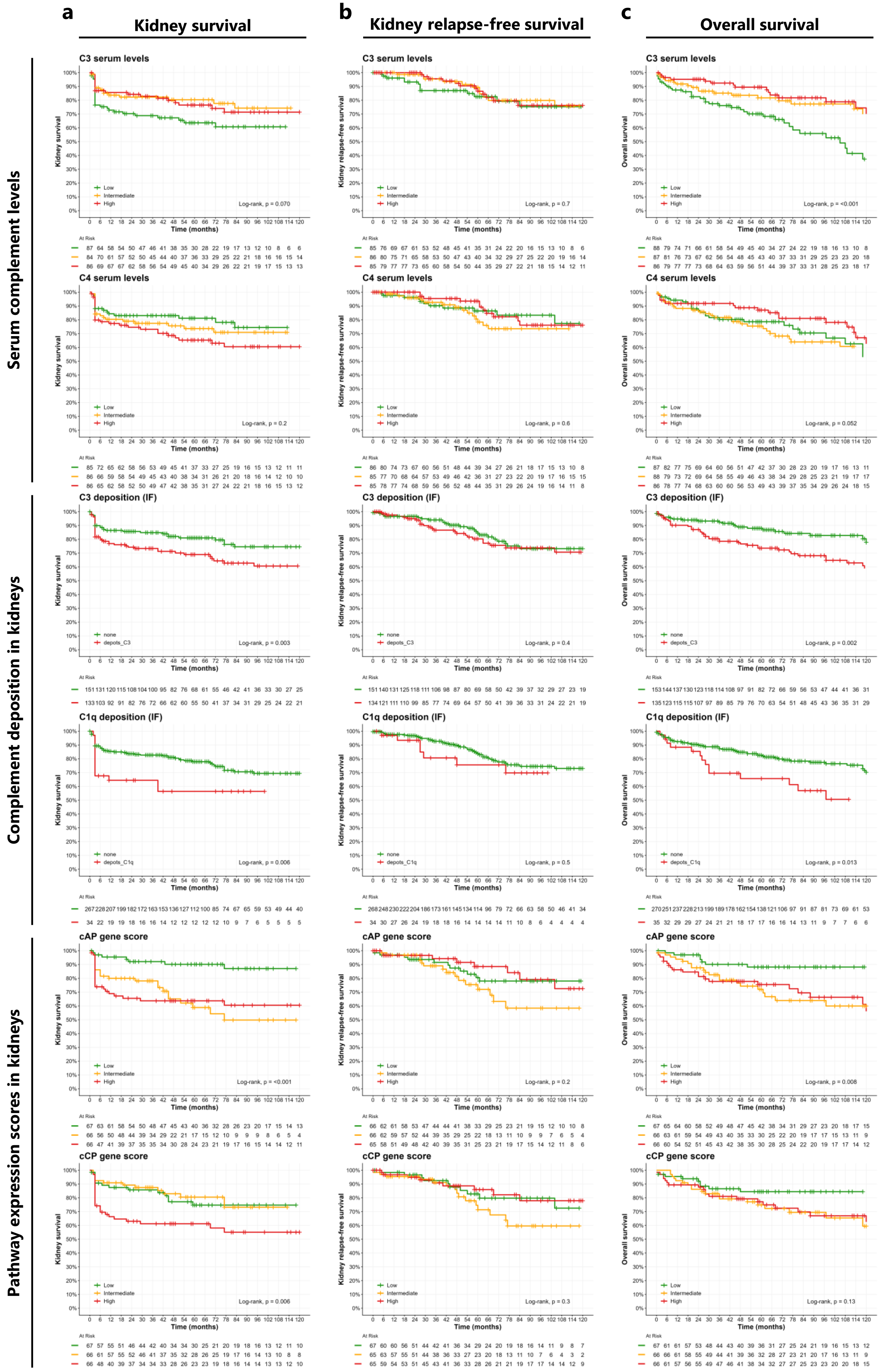

Supplementary Figure S3

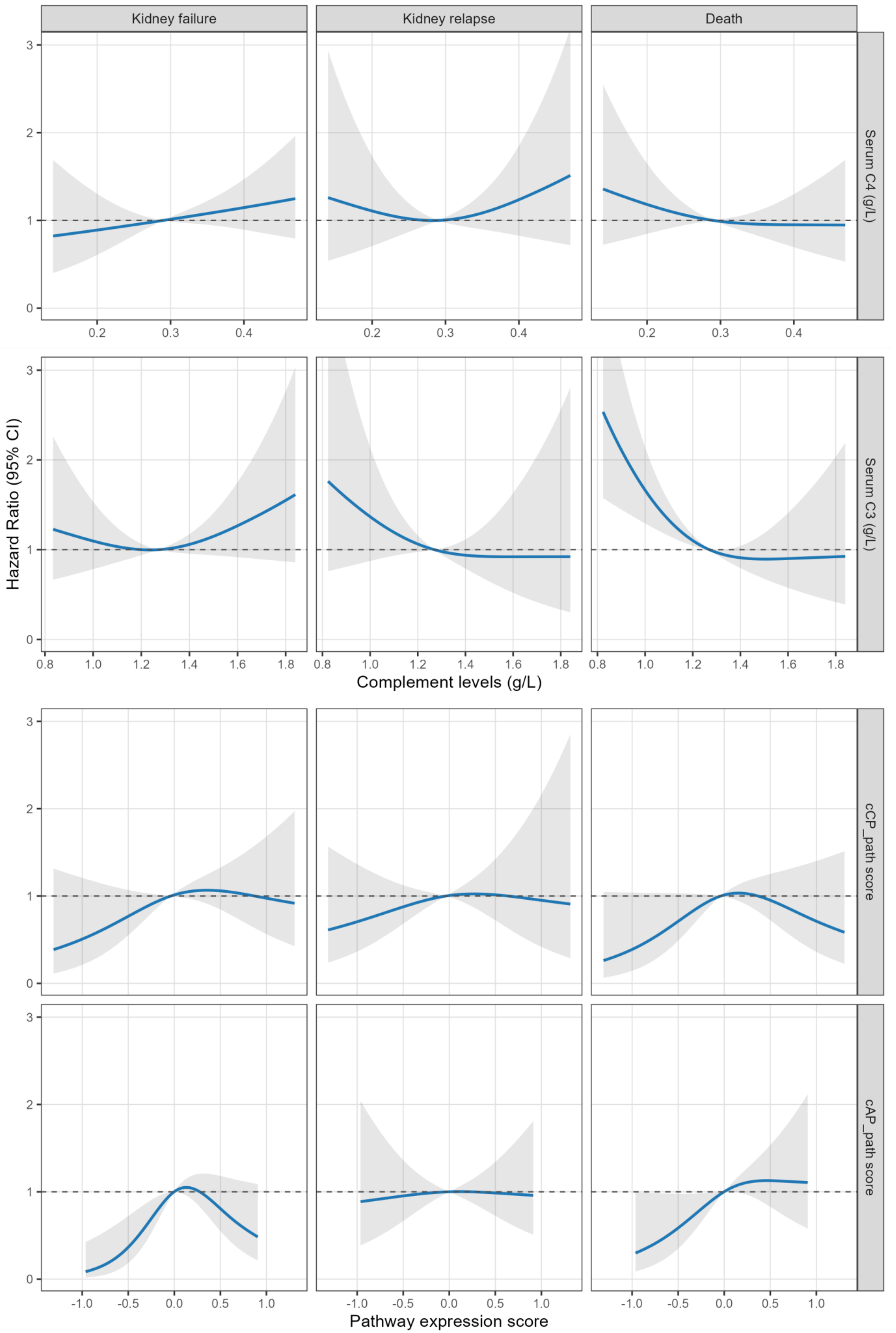

Supplementary Figure S4

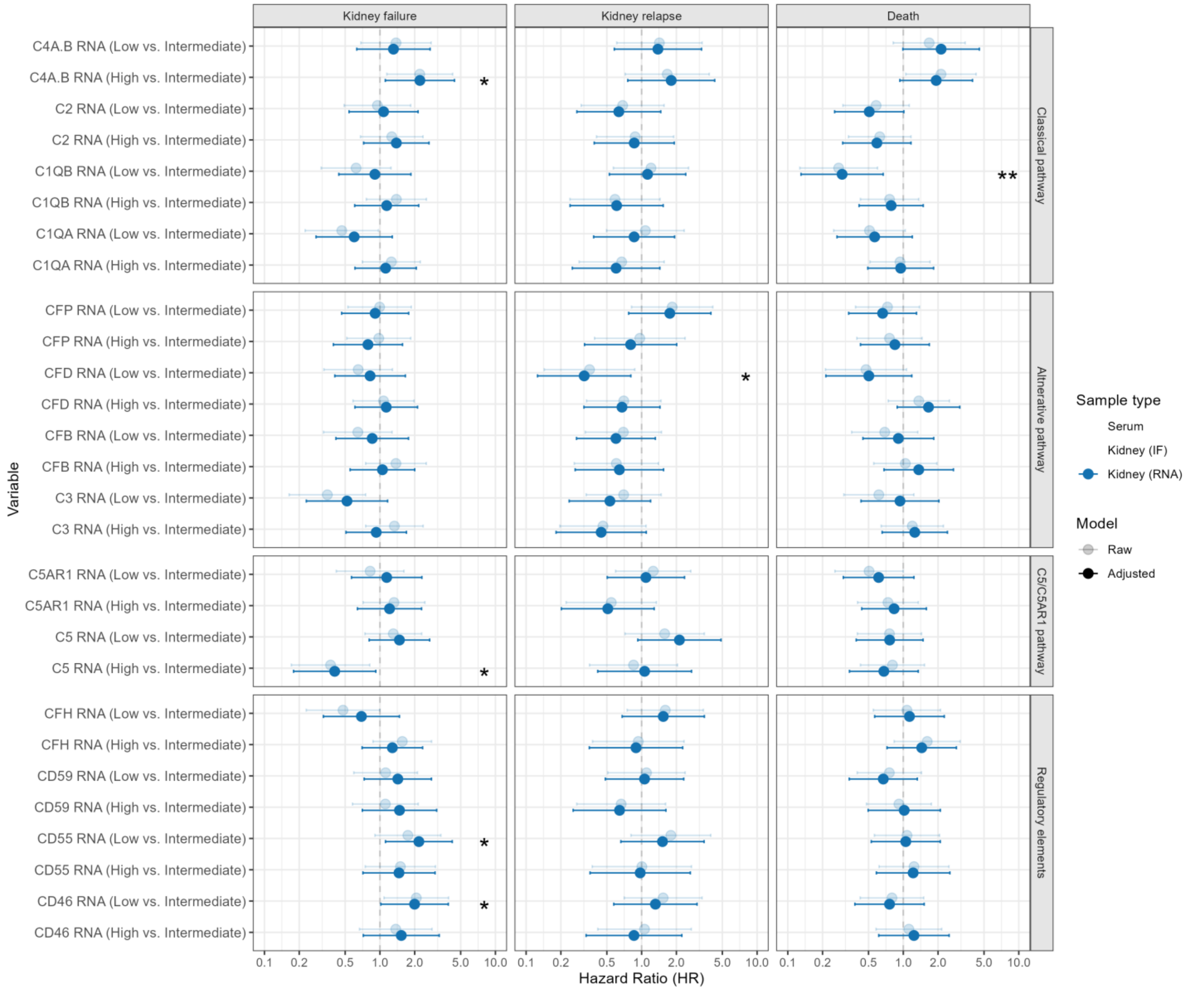

Supplementary Figure S5

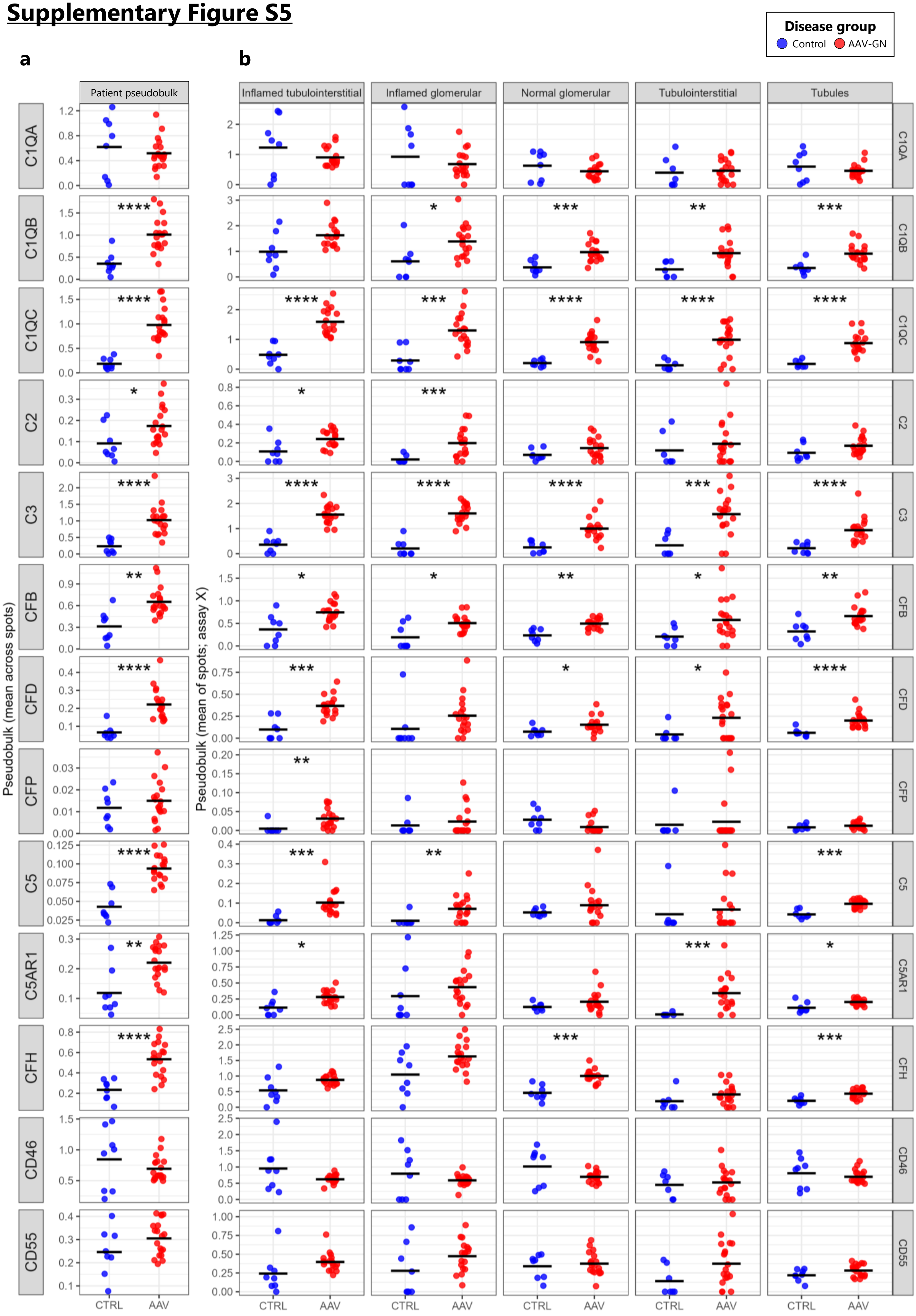

Supplementary Figure S6

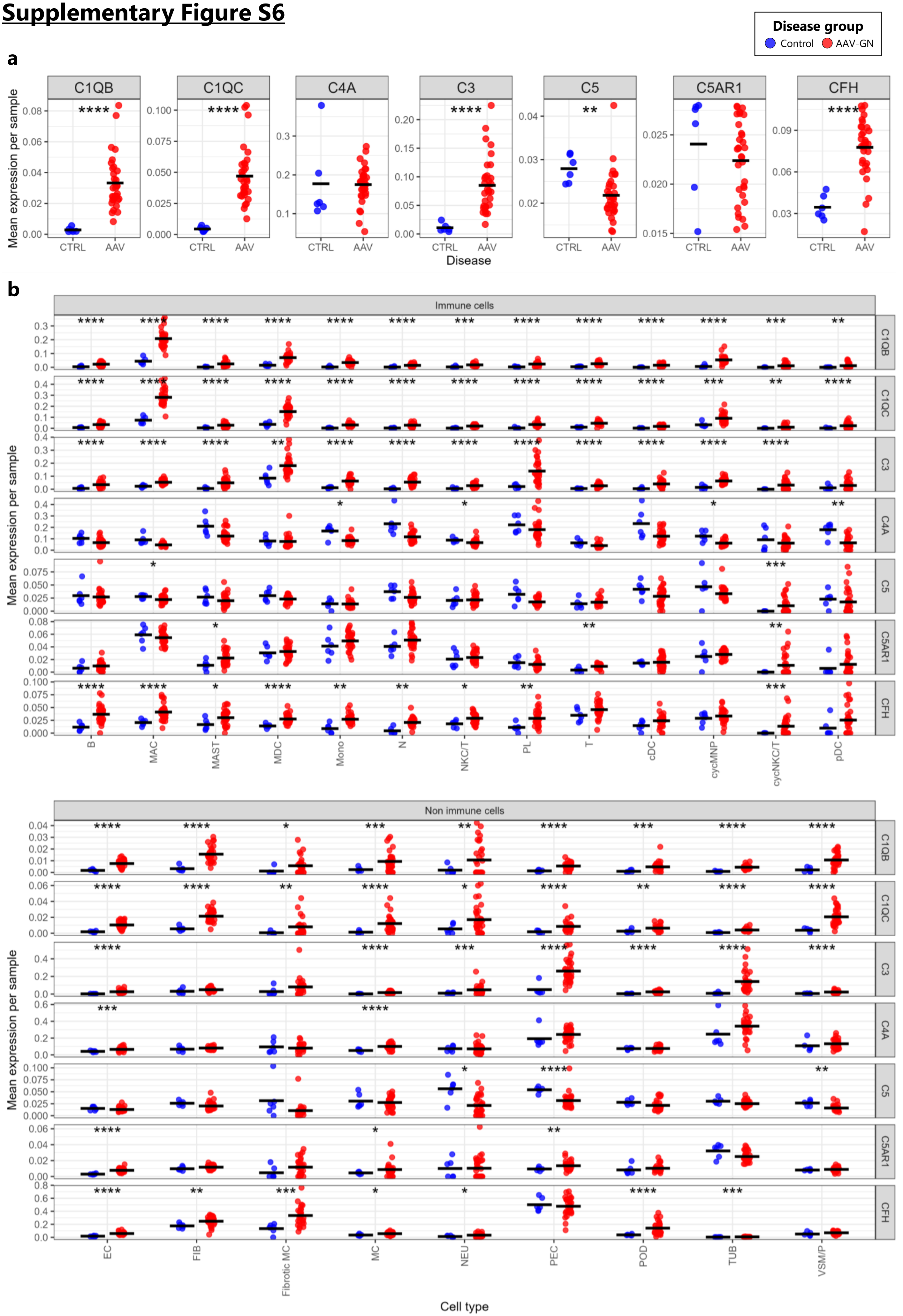

Supplementary Figure S7

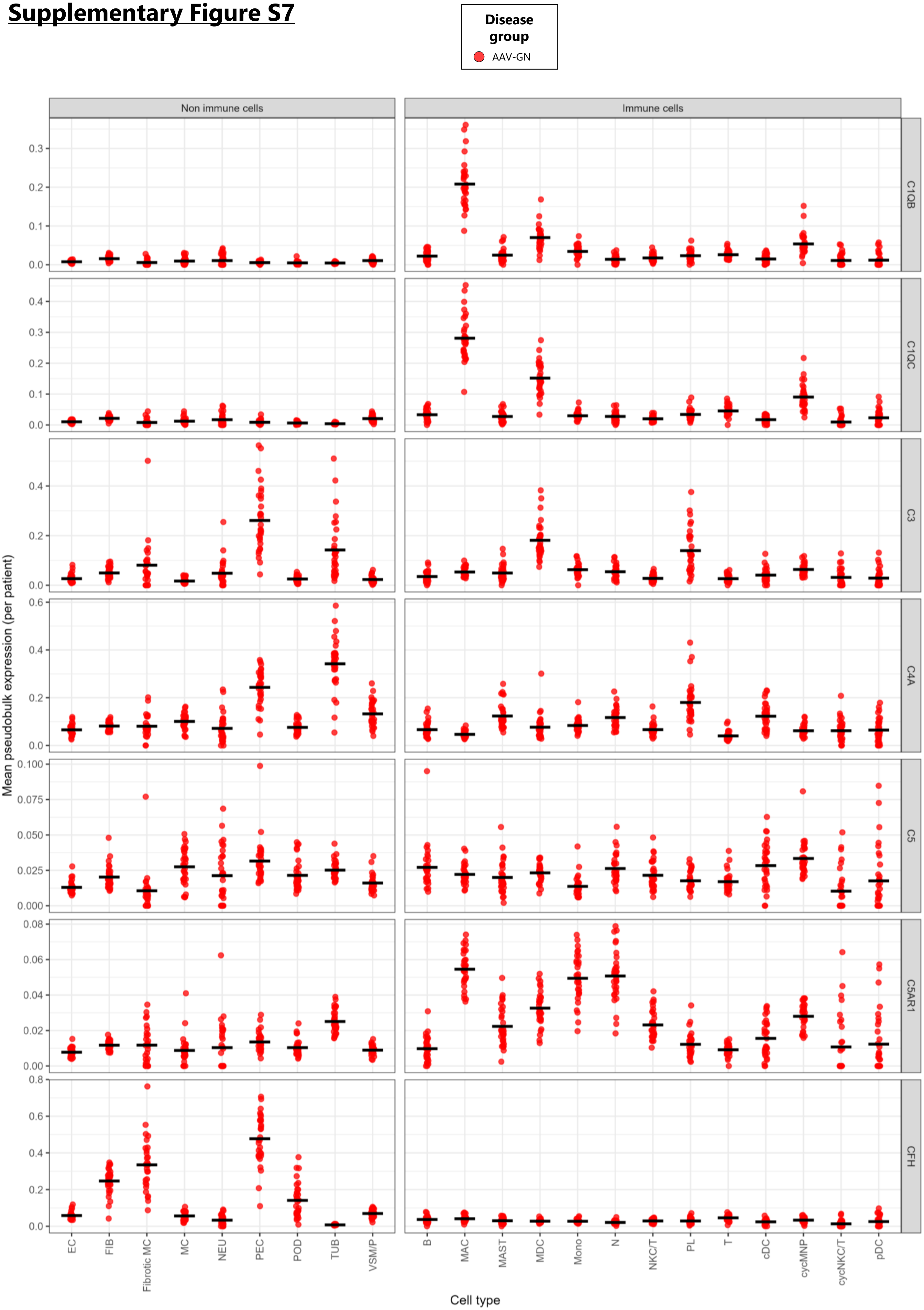

Supplement: Supplementary file 1 [file mmc1.pdf]
